# A Kinetic Model of Trp-Cage Folding from Multiple Biased Molecular Dynamics Simulations

**DOI:** 10.1371/journal.pcbi.1000452

**Published:** 2009-08-07

**Authors:** Fabrizio Marinelli, Fabio Pietrucci, Alessandro Laio, Stefano Piana

**Affiliations:** 1 International School for Advanced Studies (SISSA-ISAS) and DEMOCRITOS, Trieste, Italy; 2 Italian Institute of Technology (IIT), Trieste, Italy; 3 Nanochemistry Research Institute, Curtin University of Technology, Perth, Western Australia, Australia; Stanford University, United States of America

## Abstract

Trp-cage is a designed 20-residue polypeptide that, in spite of its size, shares several features with larger globular proteins. Although the system has been intensively investigated experimentally and theoretically, its folding mechanism is not yet fully understood. Indeed, some experiments suggest a two-state behavior, while others point to the presence of intermediates. In this work we show that the results of a bias-exchange metadynamics simulation can be used for constructing a detailed thermodynamic and kinetic model of the system. The model, although constructed from a biased simulation, has a quality similar to those extracted from the analysis of long unbiased molecular dynamics trajectories. This is demonstrated by a careful benchmark of the approach on a smaller system, the solvated Ace-Ala_3_-Nme peptide. For the Trp-cage folding, the model predicts that the relaxation time of 3100 ns observed experimentally is due to the presence of a compact molten globule-like conformation. This state has an occupancy of only 3% at 300 K, but acts as a kinetic trap. Instead, non-compact structures relax to the folded state on the sub-microsecond timescale. The model also predicts the presence of a state at 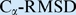 of 4.4 Å from the NMR structure in which the Trp strongly interacts with Pro12. This state can explain the abnormal temperature dependence of the 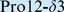 and 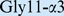 chemical shifts. The structures of the two most stable misfolded intermediates are in agreement with NMR experiments on the unfolded protein. Our work shows that, using biased molecular dynamics trajectories, it is possible to construct a model describing in detail the Trp-cage folding kinetics and thermodynamics in agreement with experimental data.

## Author Summary

Understanding the mechanism by which proteins find their folded state is a holy grail of computational biology. Accurate all-atom simulations have the potential to describe such a process in great detail, but, unfortunately, folding of most proteins takes place on a time scale that is still not accessible to routine computer simulations. We introduce here an approach that allows for constructing an accurate kinetic and thermodynamic model of folding (or other complex biological processes) using trajectories in which the process under investigation is forced to happen in a short simulation time by an appropriate external bias. An important strength of this approach is the possibility of identifying and characterizing misfolded conformations that, in some proteins, are related to important diseases. We use this method to study the folding of Trp-cage, predicting the structure of the folded state and the presence of several intermediates. We find that, surprisingly, fully unstructured “unfolded” states relax towards the folded conformation rather quickly. The slowest relaxation time of the system is instead related to the equilibration between the folded state and another compact structure that acts as a kinetic trap. Thus, the experimental folding time would be determined primarily by this process.

## Introduction

Understanding protein folding thermodynamics and kinetics is a central issue in molecular biology [1]–[3] and computer-aided modeling is becoming increasingly useful also in this field. Direct comparison between simulations and experiments requires both an accurate description of the system and the possibility to sample extensively the configuration space. In order to observe folding with molecular dynamics, it is necessary to use very large computers [4],[5], worldwide distributed computing [6], or an enhanced sampling technique [7]–[16].

A system that is almost ideal for theoretical investigation is the Trp-cage (TC5b) [17], a designed 20-residue miniprotein that folds rapidly [18] and spontaneously to a globular structure. The NMR structure (1L2Y) [17] reveals a compact hydrophobic core, in which the Trp side chain is buried. The secondary structure elements include a short 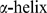 (residues 2–8), a 3_10_-helix (residues 11–14) and a polyproline II helix at the C-terminus. The folding mechanism of this system has been studied with several experimental techniques. Calorimetry, circular dichroism spectroscopy (CD) [19] and fluorescence [18] show a cooperative two-state folding behavior with transition midpoint at approximately 314 K and a relaxation time of 3.1 µs at 296 K [18]. UV-Resonance Raman [20] reveals a more complex unfolding behavior, with the presence of a compact intermediate that retains an 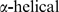 character and in which the hydrophobic core is even more compact. NMR experiments [17],[21] show a substantially cooperative thermal unfolding, but the large negative chemical shift deviations of 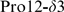 and 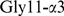 suggest that those residues might pack more tightly as the temperature is raised. Also fluorescence correlation spectroscopy experiments cannot be interpreted in terms of a simple two-state folding and the formation of a molten-globule-like intermediate has been proposed [22].

By atomistic modeling the Trp-cage folding has been studied using several different approaches [23]–[33]. In particular, with an all-atom explicit-solvent description, the folding of Trp-cage has been studied by replica exchange molecular dynamics (REMD) [31],[34]. Starting from an extended configuration, a structure with a 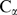 root mean square deviation (RMSD) <2 Å from the NMR reference structure is obtained after 100 ns of simulation on 40 replicas [34]. A relatively high melting temperature of 440 K is predicted. Other studies suggested that, even if Trp-cage is a rather small system, achieving statistical convergence in a REMD simulation may require much longer simulation times [35],[36]. The kinetics of Trp-cage folding was studied, in explicit solvent, by transition path sampling (TPS) [36] and transition interface sampling (TIS) [37]. The folding of Trp-cage was also investigated by two of us using the bias exchange metadynamics approach (BE) [38], in which metadynamics potentials acting on different collective variables (CVs) are exchanged among molecular dynamics (MD) simulations performed at the same temperature. Using this method it is possible to explore simultaneously a virtually unlimited number of CVs. Since all the MD simulations are performed at the same temperature the number of replicas does not grow with the system size like in REMD and in the approach of Ref. [39]. Using BE it was possible to reversibly fold Trp-cage [38], villin headpiece, advillin headpiece together with two of their mutants [40] and Insulin chain B [41] using an explicit solvent force field, in less than 100 nanoseconds of simulation with only eight replicas. Recently this method was also used for exploring the mechanism of enzyme reactions [42].

In atomistic simulations of biological systems, after an exhaustive exploration is achieved, it is necessary to extract from the trajectory the relevant metastable conformations, to assign their occupation probability, and to compute the rates for transitions among them. Several methods have been developed for this scope [43]–[48]. These methods have the big advantage of reducing a complex dynamics in a high-dimensional configuration space to a Markov process describing transitions among a finite number of metastable states. They are suitable for analyzing an ergodic molecular dynamics trajectory, but they cannot be straightforwardly applied if the system is evolved under the action of an external bias.

In this paper we present a method that allows exploiting the statistics accumulated in a bias exchange metadynamics run [38] for constructing a detailed kinetic and thermodynamic model of a complex process such as the Trp-cage folding. The approach presented here aims at extracting the same information from a BE simulation as one can obtain from the analysis of a long ergodic MD run or of several shorter runs [43]–[48]. The method relies on the projection of the BE trajectory on the space defined by a set of variables, which are assumed to describe the relevant physics of the system. These variables are not necessarily the ones that are used for the BE simulation and can be chosen 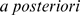. Once the CVs are selected, the rate model is constructed following three steps:

1. A cluster analysis is performed on the BE trajectories in a possibly extended CV space, assigning each configuration explored during the biased dynamics to a reference structure (bin) that is close by in CV space.
2. Next, the equilibrium population of each bin is calculated from the BE simulations using a weighted histogram analysis method(WHAM) [49] exploiting the metadynamics bias potentials.
3. Finally, a kinetic model is constructed by assigning rates to transitions among bins. The transition rates are assumed to be of the form introduced in Ref. [50], namely to depend exponentially on the free energy difference between the bins with a prefactor that is determined by a diffusion matrix 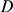 and by the bins relative position. The only free parameter in the model is 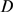, as the free energies are already assigned. Following Ref. [47] 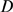 is estimated maximizing the likelihood of an unbiased MD trajectory (not necessarily ergodic).

The model constructed in this manner is designed to optimally reproduce the long time scale dynamics of the system. It can be used, for example, for characterizing the metastable misfolded intermediates of the folding process. The advantage of using biased trajectories, besides the acceleration of slow transitions, is a greatly enhanced accuracy of the estimated free energy at transition state regions.

This approach is first illustrated on the Ace-Ala_3_-Nme peptide (hereafter Ala_3_). This system is simple enough to allow benchmarking the results against a long standard MD simulation. For this system the model is capable of reproducing with excellent accuracy the kinetics and thermodynamics observed in the unbiased run. The same approach is then applied to the Trp-cage miniprotein. A model is built that allows describing the folding process, computing the folding rates and the NMR spectra, simulating a T-jump experiment, etc. The scenario that emerges is in good agreement with the available experimental data. By kinetic Monte Carlo(KMC) [53],[54] and Markov cluster analysis(MCL) [51],[52] several metastable sets (clusters) are identified. These states, except for the folded cluster, can be considered misfolded intermediates of the folding process. At 298 K two main clusters are present, with a population of 58% and 25%, respectively. The most populated is the folded state and its structural properties are very close to the NMR ensemble. The second most populated cluster retains a significant amount of secondary structure, but has a 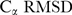 from the native state of approximately 4.4 Å. In this cluster, the Trp is trapped in a hydrophobic pocket and its distance from Pro12 and Gly11 is reduced. The presence of this cluster in the thermal ensemble of the system can explain some anomalies in the temperature behavior observed in NMR [17] and UV-Raman [20] experiments. The structures of the most populated misfolded intermediates are in good agreement with the unfolded states distances reported in Ref. [21]. Using the kinetic model a fluorescence T-jump experiment is also simulated. In agreement with the experimental results [18], a relaxation time of 2.3±0.7 µs is found. This time is primarily determined by the relaxation towards the folded state of a compact molten globule-like structure, which acts as a kinetic trap. Relaxation times among all the other clusters, including transitions between fully unstructured states and the folded state, are all in the sub-microsecond time domain. Thus, surprisingly, the relaxation time measured by fluorescence may not be directly related to the ‘folding’ transition, if one calls ‘folding’ the transition from a random coil to the native state.

## Methods

### Bin-based thermodynamic model

In the BE approach [38] a large set of CVs that are expected to be relevant for the process under investigation is chosen. A number NR (number of replica) of MD simulations (*walkers*) are run in parallel, biasing each walker with a metadynamics bias acting on just one or two collective variables. In BE the sampling is enhanced by attempting, at fixed time intervals of a few ps, swaps of the bias potentials between pairs of walkers. The swap is accepted with a probability
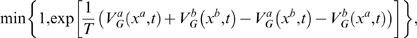
(1)where 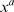 and 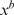 are the coordinates of walker a and b and 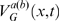 is the metadynamics potential acting on the walker a(b). In this manner, each trajectory evolves through the high dimensional free energy landscape in the space of the CVs sequentially biased by different low dimensional potentials acting on one or two CVs at each time. The results of the simulation are NR low dimensional projections of the free energy [38]. In BE the convergence of the bias potential to the corresponding free energy projection is monitored like in standard metadynamics: if the CVs are properly chosen and describe all the “slow” degrees of freedom, after a transient time, 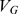 reaches a stationary state in which it grows evenly fluctuating around an average that estimates the free energy [55]. Convergence of metadynamics has been demonstrated analytically for a Langevin model [56], and numerically for several realistic systems [55], also in the presence of exchanges between different replicas [39].

Low dimensional free energy projections are often not very insightful, as in complicated processes like protein conformational transitions each minimum in a low dimensional profile may correspond to several different structures. In order to estimate the relative probability of the different structures one should find a manner to estimate the free energy in a higher dimensional space (e.g NR).

In this section a novel method to address this issue is described. The idea is to exploit the low-dimensional free energies obtained from BE to estimate, by a weighted-histogram procedure, the free energy of a finite number of structures that are representative of all the configurations explored by the system. These structures are determined by performing a cluster analysis, namely grouping all the frames of the BE trajectories in sets (*bins*) in which all the elements are close to each other in CV space. Since the scope of the overall procedure is constructing a model that describes also the kinetic properties of the system, it is important that the bins are defined in such a way that they satisfy three properties:

1. The bins must cover densely all the configuration space explored in BE, including the barrier regions.
2. The distance in CV space between nearest neighbor bin centers must not be too large. This, as it will be shown in the following, is necessary for constructing the rate model.
3. The population of each bin in the BE trajectory has to be significant, otherwise its free energy estimate will be unreliable.

A set of bins that satisfy these properties is here defined dividing the CV space in small hypercubes forming a regular grid. The size of the hypercube is defined by its side in each direction: 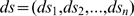 where 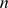 is the number of collective variables. This determines directly how far the bin centers are. Each frame of the BE trajectory is assigned to the hypercube to which it belongs and the set of frames contained in a hypercube defines a bin. This very simple approach is used here only in order to keep directly under control the distance between the bins, but the results presented in this Section apply also if the cluster analysis is performed with one of the other approaches that have been developed for this scope [43],[44],[57].

The canonical weight of each bin is estimated by a weighted histogram procedure based on the metadynamics bias potentials. The derivation that we report follows ref. [49]. Denote by 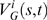 the history-dependent potential generated by the walker 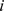 up to time 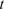 expressed in Boltzmann constant units. After a certain time 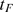 (5 ns for Ala_3_ and 22 ns for Trp-cage), metadynamics has explored all the available CV space. At the end of the simulation, an estimate of the free energy is the average of 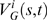 after 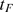 [55],[58]:
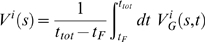
(2)where 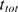 is the total simulation time. During the last part of the BE run 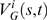 fluctuates around 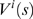 (except for an irrelevant additive constant that grows linearly with time), but these fluctuations are small if the deposition rate of the Gaussians is not excessive. In order to keep the error induced by these fluctuations under control it is convenient to consider two different bias potentials of the form of Eq. 2, one obtained extending the integral from 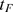 up to 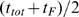, the other from 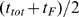 up to 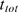. Only the configurations collected after 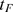 in which the two bias potentials are consistent within few 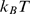 (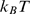 for Ala_3_ and 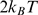 for the Trp-cage) are retained for further analysis. The unbiased probability to observe bin 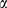 is estimated on walker 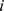 using the standard umbrella sampling reweighting formula:
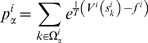
(3)where 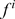 is a parameter that fixes the normalization and 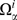 is the set of frames in the walker 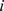 that are assigned to bin 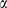 The 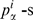 are used to construct the best possible estimate of the probability 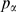 of observing bin 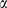. This requires estimating the error on 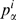. Here it is assumed that the error on a bin free energy estimate is:
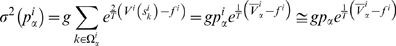
(4)where 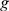 is a constant that takes into account the correlation time and
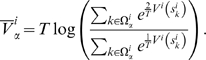
(5)


In order to simplify the notation we have neglected the position-dependence of 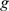. For both Ala_3_ and Trp-cage we used an upper bound for 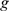 ( = 1 and 10, respectively, considering that the trajectory is saved every ps) estimated from several unbiased MD simulations started from different configurations. In the last passage in Eq. (4) the fact that 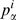 is an unbiased estimator of 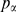 is assumed. The combined probability 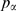 is now written as a linear combination of the 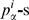, namely 
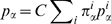
, where the weights 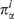 are parameters that have to be determined and 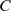 is normalization constant. The expected error on 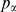 is 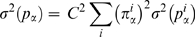. The optimal weights for each bin 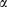 are determined separately minimizing this error with the constraint 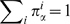. This gives 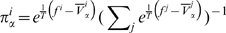 and, finally,
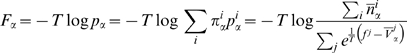
(6)with 
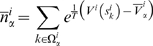
. The constants 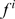 are obtained iteratively from the condition
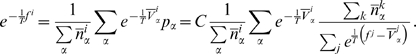
(7)


The free energy estimate given by Eq. 6 is affected by an error
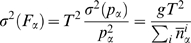
(8)consistently with what is found in the normal weighted histogram analysis method.

Within this framework, the average value of an observable 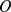 can be calculated, using the estimated free energies, as
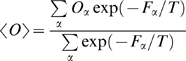
(9)where the sums run over all the bins, 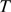 is the temperature and 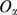 is the average value of 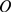 in the bin 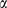. If the bin size is small enough, the bias potentials are approximately constant for the configurations belonging to the same bin [40]. Thus 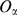 can be reliably estimated as the arithmetic average of 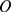 in all the configurations explored by the BE trajectory belonging to the bin 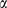. Corrections deriving from the variation of the bias potentials inside a bin have also been considered but they lead to negligible effects for small 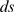.

The enthalpy 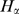 of bin 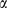 is obtained averaging the enthalpy over the structures belonging to the bin. The entropy 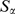 is estimated as 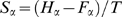. Neglecting the dependence of the entropy on the temperature, the free energy at a temperature 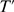 different from 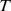 is estimated as
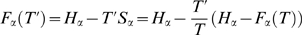
(10)with an error of 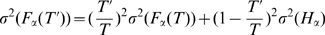.

Using Eq. 9 together with Eq. 10 allows extrapolating the average value of the observables for a few tens of K around the temperature at which the simulation is performed. The uncertainty on 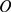 can be derived at each temperature from the error on 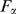, 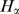, and 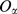 using error propagation on Eqs. 9 and 10:
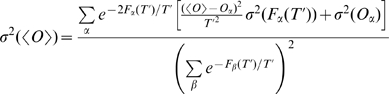
(11)where 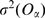 is the standard deviation of 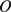 inside bin 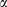.

### Bin-based kinetic model

In this section we describe a manner for constructing an approximate kinetic model describing transitions between the bins introduced in the previous Section. Constructing the model requires estimating the rates 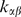 for a transition between every pair of neighboring bins 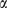 and 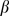. As BE trajectories are biased, the transition probabilities observed in the BE run cannot be taken as a direct measure of the true transition rates. The kinetic model is constructed assuming that the transitions between bins are described by rates of the form introduced in Ref. [47],[50], namely by diffusion with a bias determined by their free energy difference:
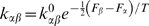
(12)where 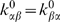 are the rates associated to simple diffusion on a flat free energy surface. This form of the transition rates ensures that the limiting probability distribution of the dynamics is correct, namely that the probability to observe bin 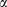 at long times scales is proportional to 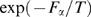. If the bins form a hypercubic grid in CV space the rates 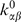 can be exactly expressed as a function of the (possibly position-dependent) diffusion matrix 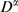 and of the hypercube side 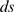 [47]. In the following to simplify the notation we denote by 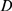 the diffusion matrix appearing in the transition rate between two bins 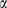 and 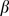 assuming that 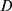 is the average of 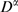 and 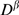 [47]. In one dimension the bins are labelled by a single integer (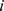) and, following Refs [47],[50], 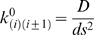 and zero otherwise. In 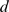 dimensions the bins are labelled by 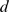 integers 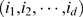. If 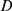 is diagonal, the one-dimensional expression for the rates can be generalized straightforwardly. If 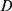 is non-diagonal the only rates different from zero are those in which one or two of the components of 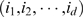 vary by one:
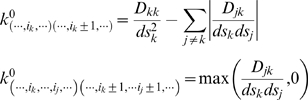
(13)This form of the rates can be derived discretizing the Fokker-Planck equation for diffusion on the regular grid defined by the hypercube centers. The derivatives are discretized as centred differences, in such a way that if 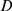 is a positive-definite matrix all the resulting rates are positive, as is required in a kinetic model. The error of this procedure scales as the square of the distance between neighbouring bins [47]. At finite grid spacing the accuracy can be improved allowing transitions between non-neighbouring bins. It can be verified that if the system is evolved with the rate equation 12 using 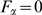, then the Einstein relation is satisfied, namely
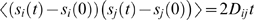
(14)


The rates given by Eq. 12 are used in a KMC algorithm [51],[52] to generate a dynamics between bins. If the bins size is small enough the KMC kinetics resembles the kinetics of an overdamped Langevin dynamics [47]. If the free energy is flat, by construction the model gives the correct diffusive behaviour but if 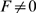 deviations from this behavior are observed when the bin size is too large. On the other hand, a small bin size can hinder the accuracy of the free energies. Thus, both large and small bin size may alter the quality of the kinetic model due to bad description of the underlying free energy surface or inaccurate sampling. Moreover even if there are no problems related to the bin size, describing the dynamics with Eq. 12 amounts to neglecting memory effects. This approximation can be particularly severe if an important variable is not included explicitly in the model. The model is expected to be reasonably accurate if the memory time is much smaller than the typical transition time (usually between metastable sets) that one wants to measure.

The diffusion matrix entering in Eq. 13 is estimated using the approach of Ref. [47], in which one maximizes the likelihood that a given MD trajectory is generated by a rate equation of the form Eq. 12. Computing 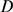 requires first generating at least one MD trajectory without the metadynamics bias. The accuracy of the procedure can be improved, if the relevant metastable states of the system are known, by running several independent MDs starting from these states. Otherwise one can select at random a few conformations along the BE trajectory and use these as the initial conditions for MD. The trajectory (or the set of trajectories) is then mapped at a time lag 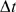 onto the bins 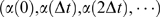. Then several KMC trajectories are run with an initial guess for 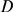, starting from the bins visited by the MD trajectory. Using the KMC trajectories one computes the conditional transition probabilities at a time lag 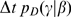 among all the pairs of bins 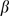, 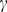 visited by the trajectory. This is evaluated by counting transitions between the bins: 
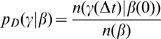
 where 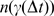 is the number of times the KMC trajectory is found in bin 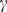 at time 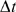 being in bin 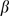 at time zero, and 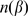 is the number of times the trajectory visits bin 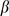. This procedure is slightly different from the one used in Ref. [47], where 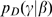 is calculated by diagonalizing the rate matrix, which in the cases considered here has a very large size (of the order of 10^5^×10^5^). The notation 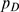 indicates that these probabilities depend parametrically on 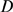.

Using these probabilities one evaluates the logarithm of the likelihood to observe the sequence of bins obtained by MD. This is given by
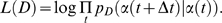
(15)

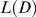
 is then maximized as a function of 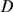. This can be done by simulated annealing, starting from an initial guess of 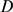 and iterating until the likelihood reaches a plateau. As outlined in Ref [48], the diffusion matrix found in this way depends in general by the chosen time lag. A common behavior is that by increasing the time lag 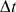 the elements of the diffusion matrix converge to a well defined value. This means that after this 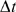 the dynamics between bins is close to Markovian and is well approximated by the model proposed. As a consequence only transition that occur on a time scale bigger than 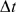 are correctly described by this model.

Applying this procedure the prefactor of the rate Eq. 12, which has the form of a jump process among a discrete set of states, is directly optimized. This is a clear advantage with respect to other methods for computing 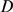, in which a continuous evolution of the collective variables is assumed. Moreover, as the free energies 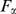 are known, the only variational parameter is 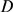 and comparably short trajectories are sufficient to determine it with a good statistical accuracy.

### Ace-Ala_3_-Nme system

The approach described in the previous two sections has been carefully benchmarked on solvated Ala_3_. For this system, it was possible to compare the predictions of the kinetic model, with the results of a very long (∼2 µ*s*) molecular dynamics trajectory.

All the BE and MD simulations were performed using the GROMACS suite of programs [59],[60] and the AMBER03 [61] force field. Ala_3_ was placed in a periodic cubic box containing 1052 TIP3P water [62] molecules. The time step was set to 2 fs and the LINCS [63] algorithm was used to fix the bond lengths of Ala_3_. The SETTLE algorithm [64] was used to fix angle and bond length of water molecules. Electrostatic and Lennard-Jones interactions were calculated with a cutoff of 1.0 nm. Lennard-Jones interactions are switched off smoothly from 0.9 nm to 1.0 nm. The neighboring list was updated every 5 steps and the cut-off distance for the short-range neighbor list was set to 1.1 nm. The Particle Mesh Ewald method [65],[66] was used to treat long-range electrostatic interactions with a maximum grid spacing for the fast fourier transform of 0.12 nm and an interpolation order of 4. A constant temperature of 300 K was achieved by coupling the system to a Berendsen thermostat [67] with a characteristic time of 0.1 ps. A constant pressure of 1 bar was achieved by coupling the system to a Berendsen barostat [67] with a characteristic time of 2.5 ps. Several independent MD simulations were performed, with a length varying between ∼30 ns and ∼30 ns, for a cumulative time of 1.8 µs.

The conformations of Ala_3_ are specified by its six backbone dihedral angles (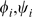, where 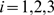) (see Fig. S1, inset). Following Refs. [68]–[70], 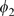 and 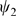 (central Ramachandran angles of Ala_3_) were considered in order to assign the main conformations of the system, denoted by 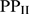 (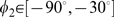, 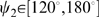), 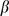 (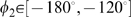, 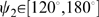), 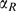 (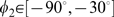, 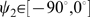), and 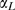 (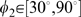, 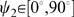). Besides the latter conformational states, eight different states were also considered in order to analyze the results of the kinetic model. These are the free energy minima with the three dihedrals 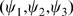 in the 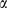 or 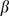 region of the Ramachandran plane, namely 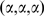, 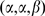, etc. (see Fig. S1).

The system was also simulated using bias exchange metadynamics (BE) [38] exploiting the six dihedral angles (see Fig. S1, inset) as CVs. Each CV was biased in a different walker. Hence, NR = 6, and each walker evolved under the action of a one-dimensional metadynamics potential acting on one of the six CVs. The width and the height of the Gaussians used in metadynamics were 0.1 rad and 0.1 kJ/mol respectively. A new Gaussian was added to the metadynamics potential every 1 ps. Exchanges of the bias potentials between pairs of walkers are attempted every 10 ps. Three independent BE simulations of 30 ns each (one simulation consist of 30 ns for each replica) were carried out in order to check the reproducibility of the results.

### Trp-cage system

The computational setup used in Ref. [38] is briefly summarized here. The simulations were performed with the GROMACS suite of programs [59],[60] and the AMBER03 force field [61], at a temperature of 298 K. The initial structure (pdb entry 1L2Y) [17] was solvated with 2075 TIP3P [62] water molecules in a 40×40×40 Å water box. The system was simulated using BE [38]. Five collective variables (CVs) were biased according to the bias exchange scheme [38]. CV1: number of 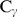 contacts; CV2: number of 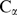 contacts; CV3: number of backbone h-bonds. CV1, CV2, and CV3 are defined as 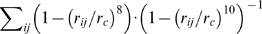 where the sum runs over the appropriate set of atoms (all the 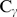 for CV1, all the 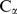 for CV2 and all the backbone H and O for CV3) and 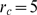, 6.5 and 2 Å for CV1, CV2, and CV3 respectively. CV4: fraction of 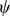 dihedrals belonging to the 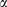 region in the Ramachandran plot, defined as 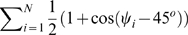. CV5: correlation between successive 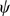 dihedrals, defined as 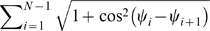. The sums in CV4 and CV5 run over all the residues. All the variables are dimensionless and none of them requires the *a priori* knowledge of the folded state. The Gaussian widths chosen for CV1, CV2, CV3, CV4, CV5 were 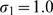, 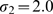, 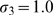, 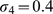, and 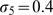, respectively. Simulations were performed with 8 walkers: one for each variable plus two walkers reconstructing a free energy surface in two dimensions: CV3-CV4 and CV4-CV5. The last walker, the “neutral walker”, is not biased by any metadynamics potential, but is allowed to exchange conformations with the others. A Gaussian of height 0.1 kJ/mol was added every 1 ps to the bias potential for all the walkers except the neutral walker. The total length of the simulations was 50 ns. In Ref. [38] it was shown that the neutral walker statistics is approximately canonical, and all the averages were there computed using only its configurations, while the trajectories of the biased walkers were not used at all. The converged free energy profiles for each walker can be found in Ref. [38]. The MD simulations used for calculating the diffusion matrix and the NMR properties were run with the same computational setup of BE simulation (except for specified changes in temperature).

#### Calculation of NMR properties

The protons chemical shift deviations (CSD) and ring current shifts (RCS) of a specific configuration were estimated using the SHIFTS program [71] version 4.1. The CSD and RCS calculated for the full ensemble of bins (or for a specific cluster), were evaluated first averaging in each bin and then averaging the result using Eq. 9 for all the bins (for all the bins belonging to a specific cluster, see Results). The RCS temperature derivatives were calculated by finite difference in the temperature interval 298–303 K. A 20 ns MD simulation starting from the NMR structure [17] at 282 K was also used for calculating NMR properties. The variation of the 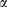 protons RCS with the temperature was calculated by applying Eq. 9 and 10.

#### Calculation of the dynamical properties: simulated T-jump experiment

The Trp solvent accessible surface area (SASA) was calculated for each bin averaging over all the configurations belonging to a bin using the program g_sas in the GROMACS distribution [72]. The Trp SASA relaxation after a temperature jump (T-jump) was estimated using the rate model. The T-jump experiment was mimicked generating 1,000,000 initial bins from an equilibrium distribution at 291 K. The bins free energies at 291 K used for generating the distribution were evaluated applying Eq. 10. Starting from each initial bin a KMC [51],[52] trajectory of 100 µs was run at 298 K. The Trp SASA was then calculated as a function of time averaging over this ensemble. The influence that the error on the free energies and on the enthalpies has on the results has been checked generating several kinetic models in which 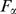 and 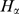 were defined adding to the original values a random number drawn from a Gaussian distribution with standard deviation given by the error interval. A simulated Trp SASA T-jump experiment was repeated for each model. The error on the relaxation time was estimated from the standard deviation of the measures on the different models.

## Results

### Application to a benchmark system: Ala_3_

Ala_3_ is a simple polypeptide that has been extensively used as a benchmark system. Although small, this system shows several protein-like features, such as intramolecular hydrogen bonds and a fragment of 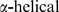 structure. Since the system is small, it is possible to characterize carefully its equilibrium and kinetic properties by extended MD simulations. In this section the results obtained by applying the approach presented in the Methods section to the Ala_3_ system will be exposed.

#### BE simulation of Ala_3_

The system was simulated using BE [38] employing the six backbone dihedral angles (see Fig. S1, inset) as CVs for biasing the dynamics (see Ace-Ala_3_-Nme system section). As expected BE improves the sampling of saddle regions (see Fig. S2B) and less stable minima (e.g. the 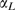 region of the Ramachandran angle). The results of the BE simulation of Ala_3_ are six one-dimensional free energy profiles (see Fig. S5), each a function of one of the six dihedral angles. After approximately 5 ns the free energy profiles do not change significantly anymore (see also Fig. S2A and S6), except for the fluctuations that are typical of metadynamics. The profiles extracted from the three independent BE runs do not show sizable differences (root mean square deviation (RMSD) of free energy ≈0.4 kJ/mol, maximum deviation ≈1 kJ/mol), and they agree with the MD results within the error bars (RMSD of free energy ≈0.8 kJ/mol, maximum deviation ≈2 kJ/mol, see Fig. S2B). The profiles obtained applying eq.2 averaging on the last 10 ns of a BE simulations are shown in Fig. S5.

#### Bin-based thermodynamic model

**Figure 1:**
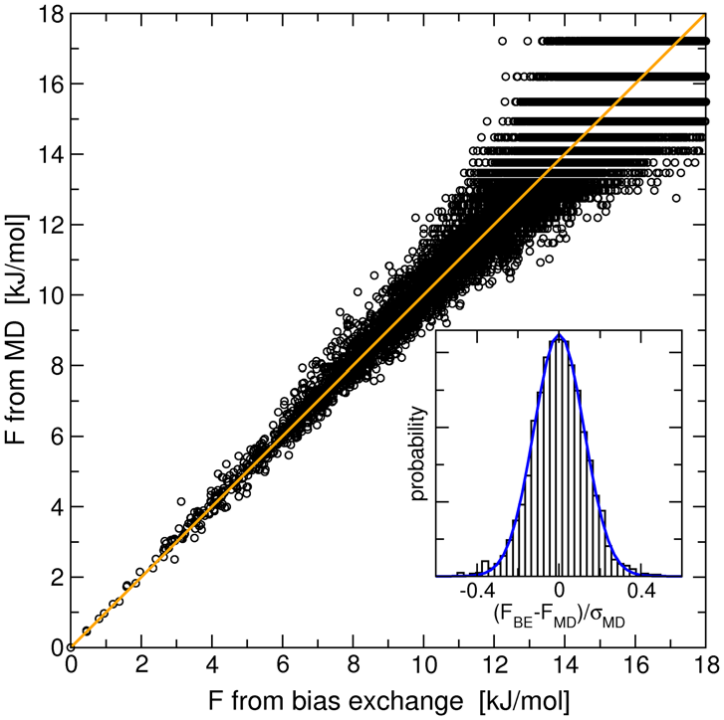
Bins free energies of Ala_3_ from BE and from MD. Correlation between the bins free energies calculated using Eq. 6 applied on BE simulations data and using the standard thermodynamics relation 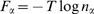 on MD results. A bin size of 30° has been used. In the inset it is shown the distribution of the deviations between the bins free energies calculated from BE and from MD, divided by the estimated error on the MD free energy. A Gaussian fit of the distribution is also shown.

Even in this simple system the different structures (see Fig. S1) are defined by the value of at least two of the six collective variables and thus one-dimensional free energies are not very insightful. In order to estimate the relative probability of the different structures we applied the approach introduced in the Methods section. The six dimensional space was divided in hypercubes of side 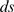 (“bins”). Due to the high dimensionality of the space the number of bins increases rapidly by decreasing the box side 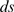. Reducing 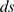 from 40° to 30° the number of bins that are visited increases from 70,000 to 300,000. On the other hand, for small 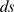 most of the bins are visited only a few times in the BE trajectories, and this hinders the accuracy of the free energy estimate (see Eq. 8). The free energy of each bin was calculated for several choices of the bins size 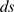 applying Eq. 6 to the BE simulation data. The free energy profile entering in Eq. 6 was calculated using eq.2 with 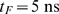. In order to reduce the error induced by the time dependent fluctuations, the bias potential was averaged independently in the two halves of the interval 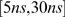 (see Methods). Only configurations collected after 5 ns in which the two averaged potentials are consistent within 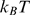 are retained for further analysis. The free energies were evaluated independently from the ∼2 µ*s* equilibrium MD trajectories by applying the standard thermodynamic relation 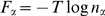, where 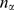 is the population of the bin 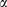. In Fig. 1, it is shown that the free energies calculated in the two manners correlate very well, especially at low free energy, where MD is accurate. Indeed, the horizontal stripes at high 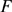 in Fig. 1 correspond to bins that are explored only a small number of times in MD. In Fig. 1, inset, it is shown the distribution of the relative error 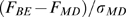 where 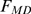 and 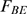 are the free energies of the bins computed by MD and BE and 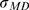 is the error on 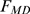 estimated by Eq. 8 on the MD trajectory (using 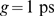). A gaussian fit to these data (blue line) shows that this relative error has an average value of zero and is normally distributed, indicating that the deviations are not systematic and are only due to inaccurate sampling. If the analysis is repeated for a larger bin size the width of the relative error distribution becomes smaller. In fact, all the bins are visited more often and the free energies are computed with better accuracy. As already underlined, in normal MD the error is small for low free energy states and large otherwise. In BE the error is instead much more uniform, and the free energy can be computed reliably also for several bins that are not even observed in MD. This property is essential for constructing a reliable kinetic model of the system.

The equilibrium population of each of the 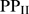, 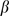, 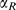, and 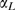 regions in the 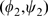 Ramachandran plot defined in the Methods section was computed by summing the populations of the bins which are contained inside. The occupation probability calculated from MD and BE simulations is reported in Table 1: extended conformations (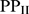 and 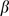) are the most populated, the helical 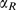 state is less populated while 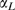 has an occupancy lower than 0.1%, in agreement with available experimental data [73]–[75] and with previous simulations [68]–[70]. Once again (Table 1), the agreement between BE and MD results is very good for all the regions.

**Table 1:** Equilibrium populations of the four main regions in the Ramachandran plot 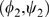 of Ala_3_.

	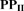	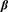	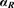	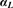
MD	34.3%	12.6%	22.0%	0.050%
BE	32.1%	12.0%	22.3%	0.085%

The results from BE are compared to those from MD.

#### Bin-based kinetic model

**Figure 2:**
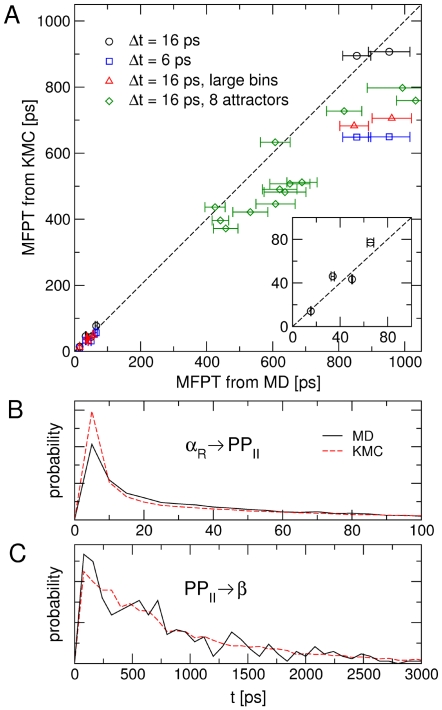
Mean first passage times between the free energy basins of Ala_3_. Panel A: correlation between the MFPT among the four regions in 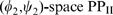, 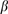, 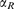, and 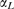, and among the eight attractors (see text and Fig. S1), obtained by MD simulations and by KMC using the kinetic model. The MFPT are calculated as the average time to go from one region to another, without passing through different regions. The error bars due to the statistical error in the MD simulations are also displayed. Large bins have a cubic side of 36°, while when not specified a cubic side of 30° is used. Panel B: distribution of FPTs from 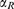 to 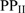 for MD and the kinetic model. Panel C: distribution of FPTs from 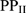 to 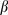 for MD and the kinetic model. For panel B and C a cubic side of 30° and a time lag of 16 ps was used for calculating the diffusion matrix 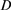.

**Figure 3:**
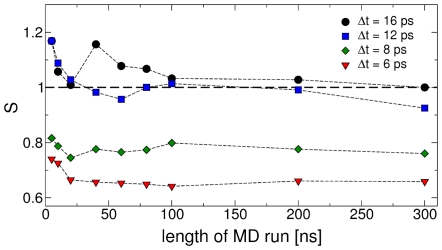
Dependence of the diffusion coefficient of Ala_3_ on the time lag and the trajectory length. Dependence of the slope 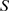 of the line fitting the pairs of mean first passage times 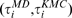 (see text and Fig. 2A) from the parameters used in the fit of the diffusion matrix 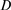: the length of the MD run and the time lag 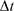. For 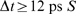 converges to the optimal value 1 (dashed line). A cubic side of 30° was used.

A kinetic model of Ala_3_ was built according to the procedure introduced in the Methods section. The free energies estimated from the BE simulations were used for constructing the kinetic model according to eq. 12. The diffusion matrix entering in eq. 13, was calculated by maximum likelihood for several choices of the time lag 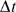 and bin size on MD simulations of length ranging from a few ns to 300 ns. To estimate the accuracy of the kinetic model the mean first passage times (MFPT) for transitions among the four regions in 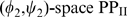, 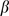, 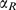, and 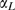 have been calculated both from MD and KMC. Moreover, the MFPT have been calculated also for transitions between the 8 bins corresponding to the 8 free energy minima obtained assigning the three 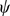 dihedral angles in the 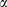 or in the 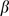 region (see Methods and Fig. S1). First, the kinetic model has been constructed for a bin size of 30° and optimizing a position independent 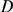 with a time lag 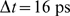. The correlation plot between MD and KMC is shown in Fig. 2A, where only transitions observed at least 50 times in the MD trajectory are reported. The overall correlation is excellent except for transitions that display a large error bar in the MD simulation. The distribution of the first passage times for well visited transitions involving the central dihedral angles are also shown in Fig. 2 (panels B and C), both for MD and KMC. The agreement is excellent especially for the 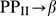 transition, which occurs on a long time scale. All these results show that the rate model is able to reproduce accurately the kinetics of the real system. In order to quantify this accuracy it is useful to consider the slope 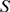 of the line fitting the pairs 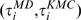 of MFPT in Fig. 2A, where 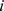 denotes a transition, as well as the RMS relative deviation
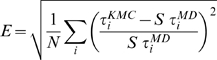
where the sum runs over the 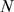 transitions. 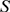 and 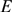, which should ideally have the values 1 and 0, have been computed for many different models in order to point out the critical issues that can affect the accuracy of the rate model:

- **The time lag** 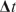 **used to estimate** 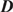. A position independent 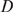 was optimized for different choices of time lag 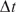 and MD trajectory length. The value of 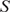 that is obtained for each 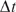 is reported in Fig. 3. For 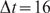 an error 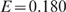 and 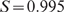 is obtained, whereas for 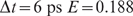 and 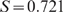, and for 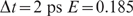 and 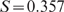. This shows that the correct time scale is obtained if the time lag 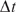 is large enough. For very small 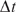 the MD trajectory cannot be approximated by a Markovian model [48].
- **The size of the bins**. Care must be taken in employing a bin size which is small enough to describe accurately the free energy of the system as a function of the CVs. Increasing the bin size from 30° to 36° still leads to reasonable transition times: the standard deviation and the slope become 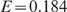 and 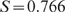 for 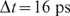 (Fig. 2A). If the bin size is further increased to 40° the kinetic model compares badly with MD: 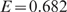 and 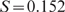. A position independent 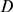 was optimized for each bin size using a 300 ns MD trajectory.
- **The length of the MD trajectory used to estimate** 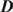 **by maximizing the likelihood**. The value of 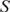 as a function of the length of the MD trajectory is reported in Fig. 3. A ∼50 ns MD trajectory is necessary to obtain a 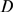 which accurately reproduces the MFPT with 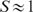. Increasing the length of the MD trajectory up to 300 ns does not change significantly 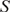, whereas employing a shorter trajectory down to ∼10 ns gives slightly larger errors. Thus changing the length of the MD trajectory between 10–300 ns affects the time scale 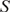 much less than the time lag 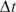.
- **The position-dependence of** 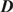. The MFPT was calculated using two different diffusion matrices obtained maximizing the likelihood only for the part of the MD trajectory that is close to two different attractors 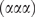 and 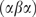, always using a time lag 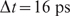. The difference in the slope 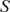 is of the order of 10–20%. This shows that the error that derives from neglecting the position dependence of 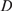 is, at least for this system, smaller than the error due to the choice of the time lag 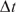.

As a general comment, even in the worst cases investigated (short 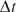, short MD trajectory), provided the bins size is not very large, the rate model produces MFPTs that are well correlated with the MD results, as shown by the relatively small value of 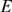. The various approximations introduced in deriving the model affect only the proportionality factor, as quantified by 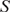, that can be ∼0.5 in the worst case (see Fig. 3). If the free energy of bins were estimated from MD and not from BE the correlation in the MFPT would be completely lost (data not shown). This is due to the fact that even in a quite extended MD simulation barriers are not well sampled; instead, in the BE simulation all the relevant bins are explored and the accuracy of the barriers between clusters is remarkably improved.

### Application to the Trp cage folding

The results presented here were obtained analyzing, with the method introduced in the Methods section the BE trajectory of Trp-cage from Ref. [38].

#### Bin-based thermodynamic model

The set of bins used for constructing the rate model was defined partitioning the five-dimensional CV space in small hypercubes according to the procedure outlined in the Methods section. A convenient choice of the cubic sides was found to be 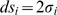, where 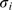 is the width of the Gaussian used for CV 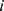. With this choice, the number of bins that are explored at least twice is ∼10000. To check the consistency of the model other cubic sides were also attempted. We checked that the CVs we are using do not lump together different conformations: indeed, the 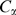 RMSD from the bin reference structure is less then 2.5 Å for most of the low free energy bins. We also verified that if a compact secondary structure element is present in the reference structure of a bin, the same structure element will be present in the overwhelming majority of frames assigned to that bin: high RMSD values are primarily determined by flexible regions that undergo fast rearrangement on the ns time scale. The free energies of the bins were estimated using Eq. 6, evaluating the biasing potentials on each of the eight replicas by Eq. 2 with 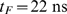. In order to reduce the error induced by the time-dependent fluctuations, the bias potential was averaged independently in the two halves of the interval 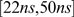 (see Methods). Only configurations collected after 22 ns in which the two averaged potentials are consistent within 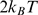 are retained for further analysis. Unlike for the Ala_3_ system in the case of the Trp-cage an extended ergodic MD simulation is not available, as equilibrating the system would require performing a run of several tens of 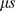. Thus, for Trp-cage it is not possible to compare the equilibrium bins free energies with the ones obtained using BE. Instead the free energies estimated with the WHAM-like [49] procedure are compared with the ones obtained using the neutral walker statistics as described in Ref. [38]. The correlation between the two free energies is excellent, especially for bins with low free energy (see also Fig. S3). As shown in Ref. [38], the neutral walker reliably reproduces the ensemble generated with normal replica exchange. This shows that the three methods, replica exchange, the neutral walker method and the weighted histogram approach described in the Methods section, all give consistent results for the statistics of the most populated bins. The errors on the free energies computed using the neutral walker ensemble are large for bins whose occupancy is low and bins of high free energy are sometimes not explored at all. The number of bins whose error is below 4 kJ/mol is approximately 1000 and 3000 for the neutral walker and the weighted histogram procedure, respectively (see also Fig. S3, inset). The weighted histogram free energies are systematically very reliable up to ∼25 kJ/mol. It is worth to note that most of the low free energy bins are visited independently by several walkers (e.g. the lowest free energy bin is visited by all the walkers).

#### Bin-based kinetic model

Like for the Ala_3_ case, the free energies of the bins were used for estimating the rate for the transitions between all the neighbouring bins according to Eq. 12. The diffusion matrix entering in eq. 13 was evaluated using the maximum likelihood approach described in the Methods section on five MD trajectories for a total time of ∼500 ns. In order to estimate the variation of 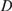 with the protein conformations, the MD trajectories were initiated from structures belonging respectively to the folded state, and clusters 2, 3, 4 and 5 (see below for the definition of the clusters). Optimizing 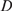 separately in each cluster leads to a cluster-dependent diffusion matrix (see Text S1). However, these variations influence the relevant observables only mildly. Indeed, the folding relaxation times (see Dynamical properties section) computed with a cluster-dependent D or with a constant D (calculated using all the MD trajectories at once) are consistent within a standard deviation of ±500 ns (see Text S1). This uncertainty is comparable to the one deriving from the error on the bins free energy (see Dynamical properties section). All the diffusion matrices, together with the relaxation times obtained using them for the kinetic model are reported in Text S1. The error bars reported for each element of the diffusion matrices indicate that they are well converged with the simulation length. As the uncertainty induced by using different 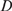 is small, all the analysis below is performed employing a position independent 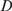 obtained by likelihood optimization using all the trajectories at once.

The maximum likelihood analysis has been repeated sampling the MD trajectory at several different time lags 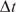. Due to important memory effects 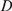 becomes approximately independent on the time lag only for 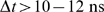. The diffusion matrix obtained with 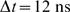 was used for constructing the kinetic model. As a consequence, the rate model is by construction unable to reproduce the kinetics of transitions that occur on a time scale shorter than 12 ns. The value of few elements of the diffusion matrix as a function of the time lag is reported in Fig. S7.

### Metastable sets (clusters) of the Trp-cage rate model

**Figure 4:**
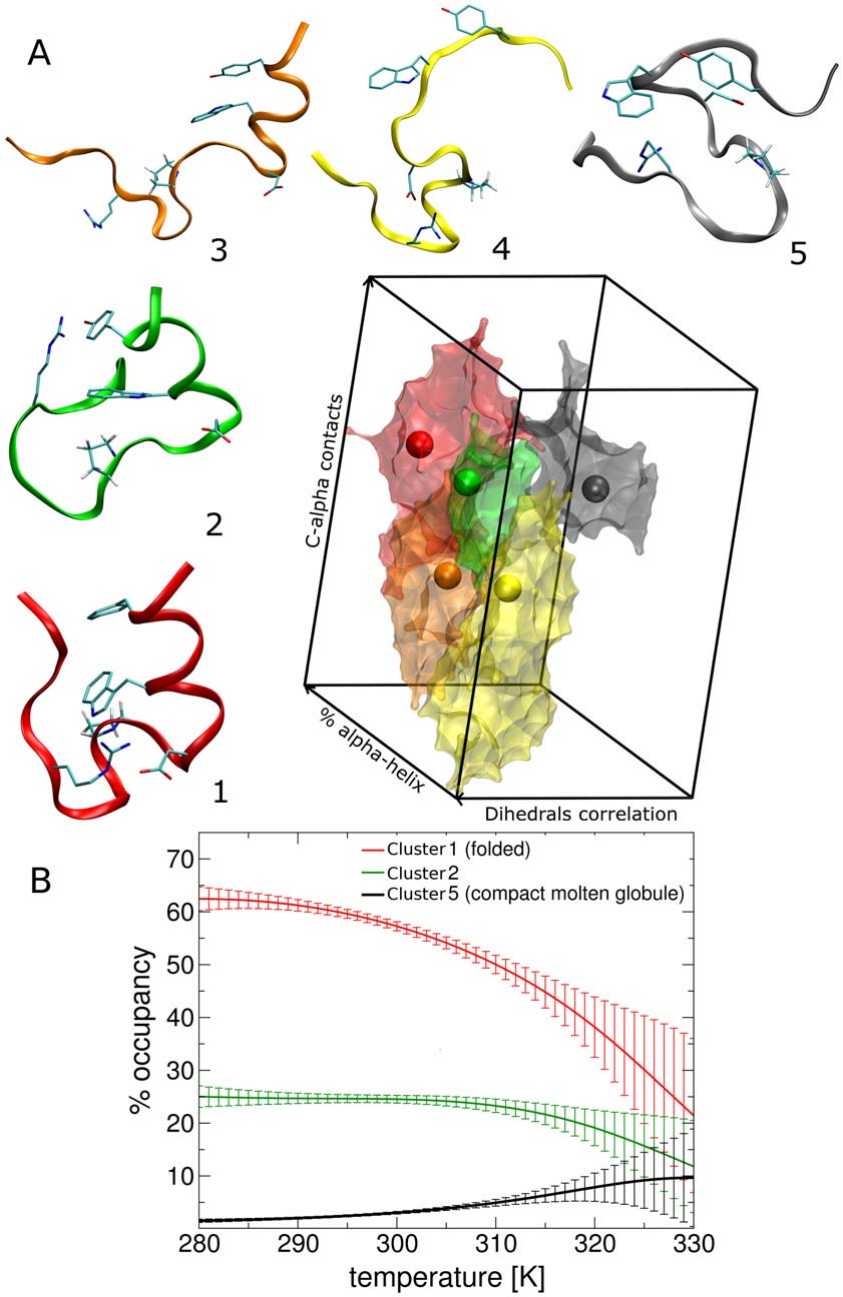
Metastable kinetic clusters of Trp-cage. Panel A: metastable sets (clusters) detected by MCL method using 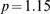. The colored spheres correspond to the lowest free energy bins of each cluster. The corresponding structures are shown with the same color code. Panel B: occupancy as a function of temperature of cluster 1, 2, and 5.

The rate model described in the Methods section has the form of a generalized rate equation with the rates given by Eq. 12. The presence of metastable sets (“clusters”) was detected applying the MCL [53],[54] method to the Trp-cage kinetic model. The algorithm requires choosing a parameter 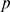 that tunes the granularity of the description: for 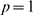 only one cluster is detected, while for large 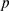 all the bins are assigned to different clusters. Several choices of the 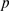 parameter are attempted (in Ref. [53],[54] the value 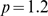 is considered). At 298 K, for 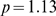 only two relevant clusters are found, one with an occupancy of ≈90% and one of ≈5%. The RMSD among the structures belonging to the big cluster is very large, indicating that, for this system, 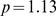 is not appropriate. For 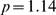 the large cluster splits in two clusters with populations of ≈12% and ≈77%. Still the larger cluster includes qualitatively different structures. At 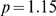 the larger cluster splits further in three, while the other clusters remain approximately unchanged. Increasing further 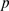 up to 1.17 does not modify significantly the three most populated clusters, whereas for 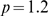 the system is fragmented in more than 10 clusters. At 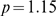, only 5 significantly populated (>1%) clusters are found, the two larger ones having a population of ≈58% and ≈25% respectively (Table 2). The average 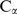 RMSD between the clusters structures and the NMR ensemble is ≈1.8 Å for cluster 1 and >4.4 Å for cluster 2 and the other clusters. Moreover, all the bins with 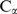 RMSD <2 Å belong to cluster 1. This allows concluding that MCL analysis using 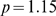 is able to identify a folded cluster with structural properties similar to the NMR ensemble. Its occupancy is of 58% at 298 K. Remarkably, at this temperature it exists another cluster with non-negligible population (25%) that contains structures that are different from the structural ensemble generated from the NMR data (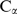 RMSD = 4.4 Å). In the next section the consequences of the existence of this second cluster in the thermal ensemble at 300 K are discussed. It is worth to note that in the MD simulations used for the calculation of 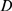, if the trajectory starts from a structure belonging to a cluster, it remains there for most of the simulation (few tens of ns). This means that MD simulations are consistent with the description of metastable states given by the MCL algorithm. In Fig. 4A, the most populated clusters obtained for 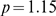 are shown using a projection on three variables, the 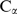 contacts, the 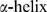 fraction, and the correlations between consecutive dihedrals. Each color corresponds to a different cluster, and the lowest free energy bin (attractor) of each cluster is depicted as a sphere of the same color.

**Table 2:** Selected properties of the Trp-cage clusters represented in Figure 4A, at 300 K.

	1	2	3	4	5
% occupancy	58.3±0.8	24.6±0.7	7.0±0.3	1.2±0.1	2.8±0.2
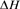 (kJ/mol)	0.0±1.9	5.0±2.6	11.7±3.8	13.8±5.3	38.2±5.3
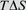 (kJ/mol)	0.0±1.9	2.9±2.6	6.5±3.8	4.1±5.3	30.7±5.3
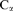 RMSD (Å)	1.82±0.05	4.44±0.03	6.76±0.04	5.54±0.06	6.08±0.05
Trp SASA (Å^2^)	47.1±0.6	70.5±1.0	126.4±0.7	116.7±1.0	140.4±0.8
Helical residues	5.31±0.02	2.91±0.03	3.86±0.04	0.66±0.03	1.70±0.03

Enthalpies and entropies are expressed with respect to the folded cluster value. The occupancy of each cluster 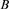 has been calculated as 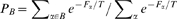 where the summation at the numerator is extended to all bins 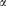 belonging to the cluster 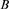. The observables reported in the table are evaluated using Eq. 9, where the summation is extended only to the bins 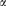 that belong to a specific cluster. The RMSD is computed as the average RMSD between the cluster structures and all the structures in 1L2Y PDB entry. The number of helical residues has been computed according to Ref. [82] using the program g_helix in the GROMACS distribution.

**Figure 5:**
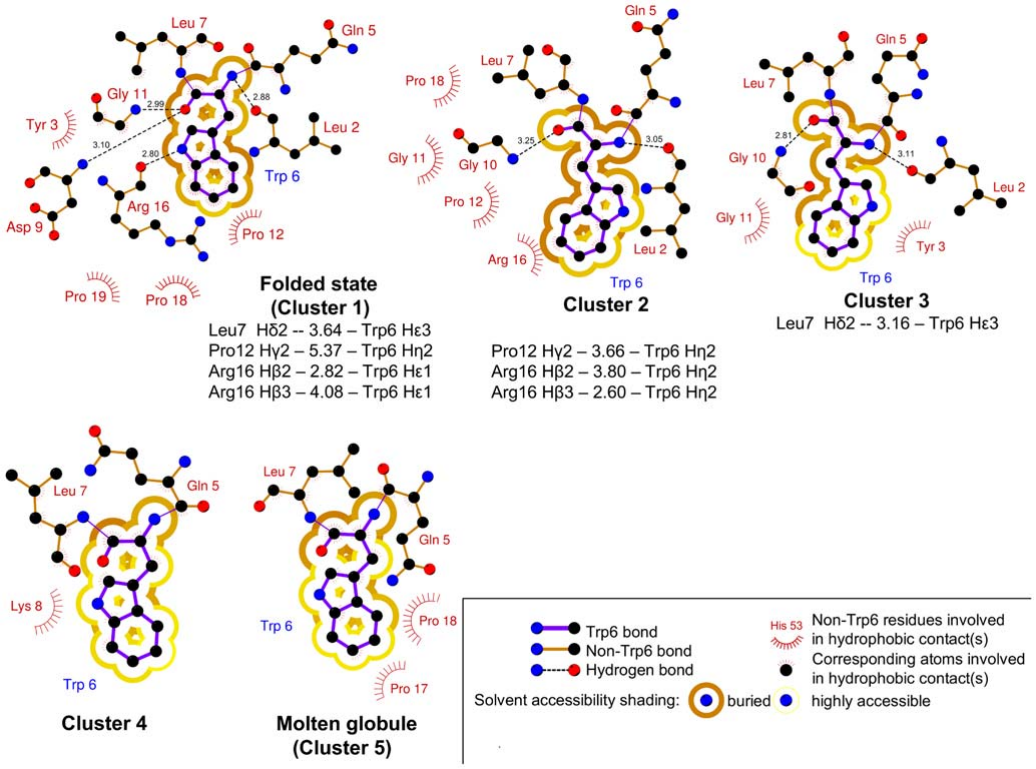
Trp6 interactions in the clusters reference structures of Trp-cage. Hydrophobic contacts within 3.9 Å and hydrogen bonds(Å) are displayed. The distances(Å) between Leu7, Pro12, Arg16 and Trp6 selected protons are shown for the 3 most populated clusters. The corresponding values can be compared with the unfolded state NOE contact distances reported in Ref. [21]. The nearest hyperpolarized Trp6 protons in the NMR experiment are selected for measuring distances. Short Ile4-Trp6 proton distances [21] (4–5 Å) are not reported in the figure since they are found mostly in open random-coil like structures and in some more compact cluster with population <1%. This figure was generated using the program LIGPLOT [81].

The properties of the clusters depicted in Fig. 4A are summarized in Table 2. In Fig. 5, the hydrophobic contacts and the hydrogen bonds with the Trp6 are shown schematically for each attractor. Selected proton distances are also displayed for the three most populated clusters. A good agreement with the NMR unfolded state distances reported in Ref. [21] is found. Cluster 1, as already anticipated, resembles very closely the NMR structure. More details will be provided in the following section (the atomic cartesian coordinates for the reference structure of cluster 1 are reported in Dataset S1). Cluster 2 has a 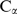 RMSD of ∼4.4 Å with respect to the NMR structure, but it retains at least part of the native 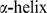. The Trp SASA in this cluster is 70.5±1 Å^2^, which compares with the value of 47.1±0.6 Å^2^ observed in the folded cluster. This indicates that Trp is shielded from the solvent also in cluster 2. Arg16 forms a 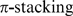 with Tyr3 (see Fig. 4A) while Trp6 is in contact with Pro12, Pro18, Gly11 and the aliphatic chain of Arg16 (see Fig. 5). As outlined in Fig. 5, except for the Arg16 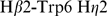 distance, the cluster 2 attractor(reference structure) shows Pro12 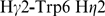 and Arg16 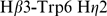 distances shorter than those in the folded cluster. The nearest hyperpolarized [21] Trp6 proton can be different in each cluster (e.g. in cluster 1 the Arg16 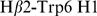 distance is shorter than Arg16 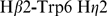). These distances are in very good agreement with those found in the NMR experiments [21] for the unfolded state. This cluster resembles the intermediate observed in a 100 ns implicit solvent simulation (Ref. [24], the atomic cartesian coordinates for the reference structure of cluster 2 are reported in Dataset S2). Cluster 3 (orange) still contains a short 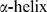. The 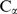 contacts are reduced with respect to the folded cluster and the Trp is partially solvent exposed. The reference structure of cluster 3 is similar to the state I of Ref. [36] and to the intermediate structure found in Ref. [31], with the difference that the Asp9-Arg16 salt bridge in cluster 3 is formed only in a fraction of the bins belonging to the cluster. This may indicate that the salt bridge is rather unstable. The Leu7 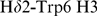 distance in the cluster 3 attractor is shorter than that in the folded state. Also in this case the distance compare well with the NMR experiments value [21]. This imply that the presence of cluster 2 and cluster 3 (the two most populated misfolded clusters) is consistent with the unfolded state ensemble information reported in Ref. [21] (the atomic cartesian coordinates for the reference structure of cluster 3 are reported in Dataset S3). The other clusters show only a small residual secondary content and can be generically referred to as “unfolded states”. The attractor of cluster 4 is stabilized by the formation of the Asp9-Arg16 salt bridge (the atomic cartesian coordinates for the reference structure of cluster 4 are reported in Dataset S4). The bins belonging to cluster 5 are mostly compact molten globule structures characterized by the presence of several hydrophobic and 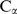 contacts (even more than in the native state) but small secondary content (see Fig. 4A and Fig. S8). In the most stable bin of this cluster Trp6 is in contact with Pro17 and Pro18 residues (see Fig. 5, the atomic cartesian coordinates for the reference structure of cluster 5 are reported in Dataset S5). In Fig. 4B the occupancies of cluster 1, 2, and 5 are plotted as a function of temperature. As expected the folded cluster (cluster 1) increases its occupancy as the temperature decreases. Its population is 50% at 310 K, a temperature that is consistent with the experimental melting point of 317 K [19],[20]. The error on the occupancies becomes large at 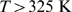, indicating that the temperature extrapolation based on Eq. 10 is unreliable after this temperature. The occupancy of cluster 5 is almost negligible at 300 K (2.8%), but it grows significantly with temperature(see Fig. 4B). The importance of this will become clear when the kinetic properties of the system will be discussed. The helical content decreases only slowly with temperature, consistently with REMD results in explicit solvent [34]. On the average, only ∼1 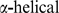 residue melts between 290 and 320 K.

### NMR Properties of Trp-cage

In order to characterize in more detail the nature of the clusters described in the previous section, it is useful to consider their NMR properties. As only cluster 1 and 2 are compact and show a significant content of secondary structure, the investigation is here restricted to these two clusters.

**Figure 6:**
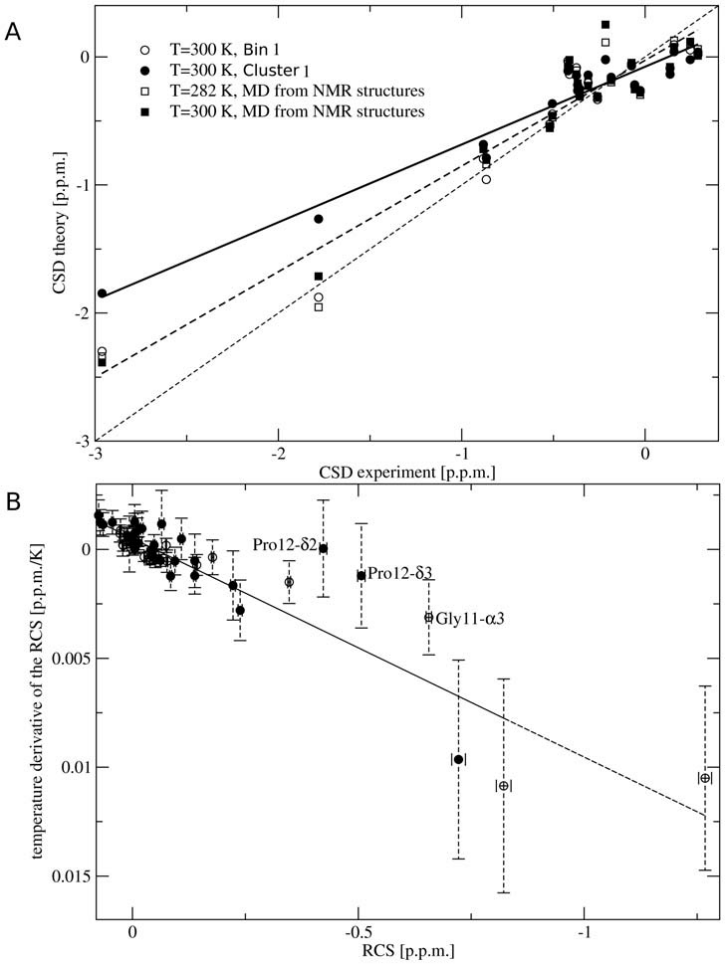
Simulated NMR chemical shift deviations and ring current shifts in Trp-cage. Panel A: correlation between experimental and calculated 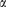 protons CSD for the cluster 1 (black circles), the lowest free energy bin (empty circles), and the ensemble obtained from a simulation started from the NMR structure at 282 K (black squares) and 300 K (empty squares). The continuous and dashed lines are obtained from a linear regression on the black circles and the squares, respectively. The thin dashed line corresponds to a proportionality factor of 1 between experiment and theory. Panel B: correlation between protons ring current shift temperature derivative and the corresponding ring current shift value evaluated at 298 K. Results are shown for 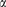 protons (empty circles) and side chain protons (black circles). Ring current shift temperature derivative is calculated as a finite difference between 298 and 303 K using the chemical shift temperature extrapolation obtained using Eq. 9 and 10.

In Fig. 6A the 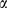 protons CSDs of cluster 1 are compared with the experimental results (full circles). The shifts are estimated as described in the Method section. The correlation between theoretical and experimental NMR CSDs is rather good (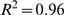), while cluster 2 shows a much smaller correlation with experiments, especially for protons that have negative CSDs. The correlation with NMR data is even smaller for all the other clusters. This confirms that the cluster classification deriving from Markov cluster analysis accurately discriminates between the folded state (cluster 1), an unfolded state with several native-like features (cluster 2), and all the rest. The correlation with experiments is retained using in the average the full ensemble of bin (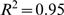).

Even if correlation is good, it has to be noted that the proportionality factor between theoretical and experimental CSDs is 0.46 in the full ensemble of bins and 0.6 in cluster 1. To investigate the origin of the variations in the proportionality factor two 20 ns equilibrium MD simulations have been performed, at 282 K (experimental temperature) and at 300 K, starting from the NMR structure and with the same computational setup used in the BE simulation. At both temperatures the proportionality factor with experimental CSDs is 0.8 instead of 1, therefore 0.8 has to be considered the reference value for our computational setup. The optimal proportionality factor of 0.8 is obtained if the CSDs are computed on the lowest free energy bin of cluster 1. The slope difference between 0.6 (cluster 1) and 0.8 may be ascribed to small inconsistencies between the ensemble of structures generated with BE and by an unbiased MD starting from the NMR structure. The further slope variation when the calculation is extended to the full ensemble of bins is most likely a consequence of calculating NMR properties at 298 K instead of at the experimental temperature of 282 K where the population of cluster 1 is larger.

Using a similar procedure (see Methods) RCS and its temperature derivative were also computed. It is worth to note that most of the large CSD are due to the Trp RCS [17]. The protons whose RCS is large are also those whose RCS depends more strongly on 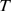, in excellent agreement with the experimental data [17]. The 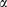 protons RCS temperature derivatives as a function of the RCS are plotted in Fig. 6B. The results are plotted as a function of the RCS estimated at 298 K. The comparison is performed at 298 K and not at the experimental temperature of 282 K in order to avoid error propagation that is unavoidable if Eq. 10 is used for extrapolating the results for a large temperature difference. Despite of this, the two observables correlate linearly (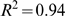 for the 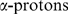), consistently with experiments [17]. Side chain protons in the C-terminal part of the protein fall on the same correlation line, also in agreement with the experiments [17]. A few protons deviate significantly from this linear behavior. The most significant deviation are observed for 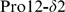, 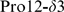, and 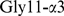, the last two being also reported experimentally [17]. The RCS of 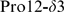 and 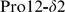 is large, while their RCS derivative is almost zero. The cluster decomposition proposed here can be used to elucidate the presence of these outliers. In fact, the RCS of 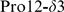 is −0.53±0.01 p.p.m. and −0.97±0.02 p.p.m in cluster 1 and 2 respectively, while other protons (except 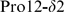 and 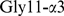) have RCS which are less negative in cluster 2 than in cluster 1 or similar in the two clusters. The RCS of 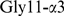 has a similar value in both clusters. This significant difference derives from the fact that 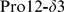 and 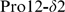 in cluster 2 are much closer to Trp than in cluster 1. Since, increasing the temperature, the relative population of cluster 2 and 1 changes (see Fig. 4B), the RCS of 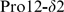, 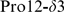 and 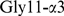 changes with temperature less than the RCS of other protons. In view of these results, the anomalous behavior of 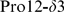 and 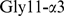 observed experimentally can be considered a signature of the presence of cluster 2 in the thermal ensemble of Trp-cage.

### Dynamical properties: simulated Trp SASA T-jump experiment

The fluorescence relaxation after a temperature jump (T-jump) was estimated according to the procedure outlined in the Methods section. This observable is used in Ref. [18] to infer information on the Trp cage folding kinetics. The fluorescence properties of the system are here estimated by computing the Trp SASA, which is known to correlate with fluorescence [76]. The result shows a smooth decay to an asymptotic value on the time scale of the microseconds. A double exponential decay model describes very accurately the data (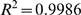, see Fig. S4). The two time constants are 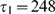, and 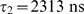. The large gap between the first and the second time constant is a strong indication of two-state behavior. The value of 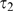 is in agreement with the experimental relaxation time of 3.1 µs for the florescence T-jump [18]. This shows that the rate model is capable of reproducing accurately the dynamics of the real system, at least for what concerns the relaxation of fluorescence. The microscopic rearrangements that determine 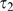 will be discussed in detail in the next section. The influence that the error on the free energies and on the enthalpies has on the results is ∼500 ns (see Methods). The error deriving from neglecting the position dependence of 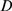 is ∼500 ns (see section Application to the Trp cage folding and Text S1). Thus the overall error on the relaxation time is 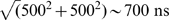. Including the correction suggested in Ref. [77] to take into account the unphysical viscosity of TIP3P water [78] the relaxation time is 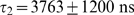, still in fair agreement with experiments.

### Trp-cage folding dynamics

**Figure 7:**
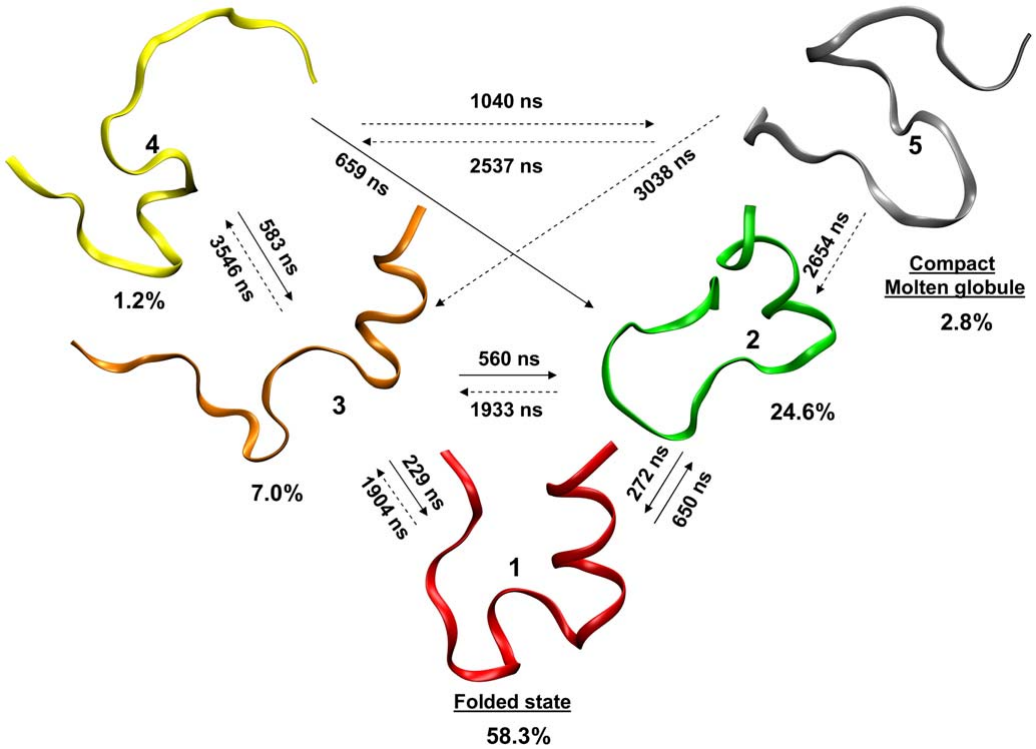
Schematic representation of the Trp-cage folding dynamics. Times (inverse of rates) for the transitions between the relevant clusters are shown on the arrows. The uncertainty on each transition time due to both the error on the free energies and the position-dependence of 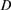 is at most 40%. Only the clusters whose population is higher than 1% are shown. Continuous arrows correspond to direct transitions between clusters that occur on a time smaller than 1 µs. Dashed arrows correspond instead to transition that occur on a time larger than 1 µs or taking place through other intermediate low-populated clusters, not represented in the Figure.

Here the dynamics of the system is investigated in more details, still using the rate model introduced in the Methods section. The characteristic times of the system are related to the eigenvalues of the rate constant matrix. Consistently with what is found for the Trp SASA relaxation, the second largest eigenvalue corresponds to a characteristic time of 2447 ns. The third eigenvalue corresponds to 434 ns, with a gap of 2013 ns from the first, consistently with a two state behavior [18]. The second eigenvector has large positive components in cluster 1 and 2 and large negative components in cluster 5. This suggests that the longest relaxation time of the system is associated to a transition between these states. In order to analyze more quantitatively this issue, the rates for the transitions between the clusters found by Markov cluster analysis were extracted from a very long KMC simulation (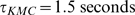). For two clusters A and B with occupancy 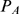 and 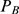, the rate constant to go from A to B was calculated counting the number of times 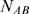 that a trajectory goes from A to B without passing from any other cluster during the KMC simulation. The rate to go from A to B was estimated as 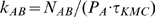. To minimize the number of recrossing, the KMC trajectory is assumed to visit a cluster any time it visits any bin belonging to the group of lowest free energy bins containing 70% of the cluster population. Bins that do not fall in this definition were considered as transition states. The transition rates obtained in this manner are represented in Fig. 7. For clarity, all the clusters whose occupancy is below 1% are omitted from the figure. The equilibration between cluster 1 and 2 is rather fast and transition times to cluster 3 are also in the sub-microsecond domain, but when the system reaches cluster 5 on average ∼2 µs are necessary to return to the folded cluster. The folding pathways schematized in figure are consistent with the two routes proposed by Ref. [36], except for the transitions involving cluster 5. The folding pathway initiating from cluster 4 and passing from cluster 3 is characterized by the early formation of an 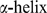 and resembles the pathway passing from state I in Ref. [36]. The pathway passing from cluster 2 is instead characterized by the formation of several hydrophobic contacts, while the 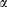 content remains on average lower. This resembles the pathway passing from state L in Ref. [36]. If the molten-globule state (cluster 5) is neglected the folding and unfolding rates are compatible with those reported in Ref. [37], considering the difference in the force field.

## Discussion

### The kinetic model and its validation

The approach presented here exploits the trajectories of multiple metadynamics simulations for building a thermodynamic and kinetic model of complex processes (e.g. protein folding) whose description requires a large number of collective variables. The aim of the model is to reproduce the long time scale dynamics of the system and to extract the metastable sets (clusters) of the kinetic process. These states may correspond, for example, to misfolded conformations. The model is constructed as follows: in a first step the equilibrium probabilities of a finite set of conformational states, or bins, are determined by a weighted-histogram procedure exploiting the low-dimensional free energies estimated by metadynamics. In a second step an approximated description of the kinetics is obtained estimating the transition rates among the bins. The diffusion matrix entering in the model is estimated by a maximum-likelihood procedure [47] employing relatively short unbiased MD trajectories. The approach was tested on the Ace-Ala_3_-Nme peptide in explicit solvent using the six backbone dihedral angles as CVs. For this system equilibrium MD trajectories on the microsecond timescale are sufficient to sample the relevant conformational space and were used as a reference to evaluate the accuracy of the kinetic model obtained from the BE results. The bins free energies obtained with the method presented here are in excellent agreement with free energies computed from equilibrium MD. The transition rates among neighboring bins are used to run a long KMC. The mean first passage times among selected states obtained in this way are in agreement with those extracted from the reference MD simulations.

### A kinetic model of Trp-cage folding

Trp-cage is a designed miniprotein that, due to its small size and fast folding rate, has been the object of several theoretical investigations. Here this system is analyzed with a new method, introduced in this paper, that allows deriving a kinetic model of the system by analyzing a set of biased MD trajectories. The model shows the presence of several metastable states (clusters). The most populated one can be classified as the folded state. The second most populated cluster has a 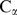 RMSD of ∼4.4 Å from the NMR structure and retains part of its secondary structure (see Fig. 4A). In this cluster the Trp is more strongly packed between Gly11 and Pro12 than in the NMR structure and its population relative to cluster 1 increases with temperature (see Fig. 4B). This can explain the anomalous behavior of the temperature dependence of the CSD of 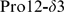 hydrogen atom observed both experimentally [17] and in the simulated NMR experiment (see Fig. 6B). The cluster 2 and cluster 3 reference structures are consistent with experimental unfolded state distances [21] (see Fig. 5). The presence of these two clusters is also in agreement with the strengthening of proline(s)-Trp excitonic interactions with temperature and the broad 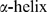 melting observed in Ref. [20].

In spite of the presence of several intermediates both the simulated T-jump experiment (see Fig. S4) and the spectrum of the kinetic matrix associated with the rate model are consistent with a two state kinetics [18]. The calculated time constant of the folding process is ∼2.3±0.7 µs (or ∼3.8±1.2 µs including the correction of Ref. [77]) in fair agreement with the experimental relaxation time [18]. To investigate the folding dynamics using the kinetic model we derived a folding mechanism which involves the detected intermediates (see Fig. 7). Starting from open structures, the folding process can follow two main routes. One of them consists in an earlier formation of the N-terminal 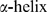 (cluster 3) followed by the hydrophobic collapse, while the other involves first the formation of hydrophobic contacts with less helical content (cluster 2) and then the completion of both secondary and tertiary structure. This is in agreement with the pathways found in Ref. [36]. The time required to undergo these transitions is in the sub-microsecond time domain, which is less than the slowest relaxation time found in the simulated T-jump experiment and more consistent with the third eigenvalue of the kinetic matrix. Indeed, the folding mechanism (see Fig. 7) shows that, if Trp-cage reaches the molten globule state, more than 2 µs are necessary to reach the folded state. This implies that the experimental folding time is ultimately determined by the slow equilibration between the first two clusters and the compact molten globule state that acts as a kinetic trap. In this state no secondary structure element is present, but a hydrophobic core with several tertiary contacts is formed. In Ref. [79] the Pro12Trp mutation brings to an increased stability of the folded state and a faster folding time of ∼1 µs. This seems to be in agreement with the folding mechanism presented here, since the mutation would strongly stabilize cluster 1 and cluster 2 but not the molten globule cluster. A possible way to assess experimentally the presence of the molten globule could be a mutation of Pro17 to a more polar residue (e.g. Asn) or a chemical modification of this residue as the lower rigidity associated to the absence of the Pro17 ring could destabilize the folded state [80]. In fact in the attractor of cluster 5 Pro17 shows a strong interaction with Trp6, and this interaction does not play a key role in other relevant clusters (see Fig. 5).

In conclusion, we have presented an approach aimed at constructing a rate model for complex biomolecular processes starting from a set of biased MD trajectories. One could argue that other approaches aimed at the same purpose are based on less severe assumptions. Distributed simulation techniques allow computing the folding rates directly, and have been applied successfully for studying folding in explicit solvent of even larger systems [32],[43],[45]. Normal replica exchange [29],[34], when converging, provides a direct measure of the equilibrium distribution, and does not require a complicated reweighting procedure. Finally, if one would use an implicit solvent description of the system, one could observe several folding/unfolding events by simple finite-temperature molecular dynamics, and it would not be necessary to use an enhanced sampling technique. In this framework, a rate model for the system could be constructed in a more rigorous manner [43],[44],[46]. Still, despite of the approximations that are done, the approach presented here provides a picture of the dynamics and thermodynamics of the system that is detailed and in agreement with all the experimental evidences presented so far. We believe that this result ultimately derives from the combined use of an accurate (but expensive) force field, and of a method that, at the price of generating non-equilibrium trajectories, allows an efficient exploration of configuration space and the accurate calculation of free energies.

## Supporting Information

Dataset S1 Cartesian coordinates of folded state (cluster 1) reference structure in Protein Databank format.
(0.02 MB TXT)

Dataset S2 Cartesian coordinates of cluster 2 reference structure in Protein Databank format.
(0.02 MB TXT)

Dataset S3 Cartesian coordinates of cluster 3 reference structure in Protein Databank format.
(0.02 MB TXT)

Dataset S4 Cartesian coordinates of cluster 4 reference structure in Protein Databank format.
(0.02 MB TXT)

Dataset S5 Cartesian coordinates of cluster 5 (compact molten globule) reference structure in Protein Databank format.
(0.02 MB TXT)

Figure S1 Structures of the attractors for the relevant free energy basins of Ala3 found in the MD and BE simulations. Inset: Schematic picture of Ala_3_ test system. The dihedral angles φ and ψ displayed in the figure are chosen as CVs for the BE simulation. They are labeled with suffix according to their position along the chain.
(4.96 MB TIF)

Figure S2 Free energy profiles as a function of φ1 (see Fig. S1) for Ala3. Panel A: time evolution of −V_G_(s,t) during a BE simulation between 1 and 8 ns; after ∼5 ns the bias potential converges and grows parallel to itself. Panel B: Free energy profile from the 1.8 µs MD simulation compared with the profiles obtained from three independent BE simulations. The 3 BE profiles are obtained by applying eq. 2.
(0.49 MB TIF)

Figure S3 Correlation between free energies of neutral walker and WHAM for Trp-cage. Correlation between the bins free energy evaluated using the approach described in the Methods section and using the neutral walker ensemble at T = 298 K. Inset: cumulative number of bins with an error smaller than the value reported in abscissas. The error is estimated using Eq. 8. The value of g entering this equation is estimated from the correlation time of the bin occupancies and is equal to 10 ps.
(0.39 MB TIF)

Figure S4 Simulated Trp-SASA T-jump of Trp-cage. Simulated TRP SASA evolution as a function of time at 298 K starting from an initial distribution at 291 K (black line). The red line is a double exponential fit to the data. The two time constants of fit are τ_1_ = 248 ns, τ_2_ = 2313 ns. The diffusion matrix entering in the kinetic model was calculated using several MD simulations for a cumulative time of ∼500 ns. A time lag of 12 ns was used in the maximum likelihood approach for calculating D.
(1.16 MB TIF)

Figure S5 Free energy profiles of Ala3 along the six backbone dihedral angles. The profiles are calculated using eq. 2 on the last 10 ns of a 30 ns BE simulation.
(0.15 MB TIF)

Figure S6 Free energy profiles as a function of time for Ala3 obtained with a 30 ns BE simulation. −V_G_ is reported for each backbone dihedral angle at several times after the filling time. Each time is represented with a different color: black (10 ns), red (11 ns), green (12 ns) and blue (13 ns). The parallel growth in time of the metadynamics bias potential is evident from the picture.
(0.36 MB TIF)

Figure S7 Diffusion matrix of Trp-cage as a function of the time lag. Few elements of the diffusion matrix are reported. A MD trajectory of ∼500 ns and the maximum likelihood approach explained in the manuscript is used for calculating D at each time lag. After approximately 8–10 ns the diffusion matrix elements show a converging behaviour.
(1.00 MB TIF)

Figure S8 Bins network topology at T = 298 K projected on three dimensions: Cα contacts, dihedral correlations and α-helix fraction. Each bin is represented as a sphere whose dimension and color is associated with the free energy (kcal/mol). The location of the folded state and the molten globule (cluster 5) lowest free energy bins are indicated in the figure.
(3.07 MB TIF)

Text S1 Diffusion matrix tables and correspoding rates.
(0.07 MB PDF)

## References

[1] Shea JE, Brooks CL (2001). From folding theories to folding proteins: A review and assessment of simulation studies of protein folding and unfolding.. Annu Rev Phys Chem.

[2] Plotkin SS, Onuchic JN (2002). Understanding protein folding with energy landscape theory – Part I: Basic concepts.. Q Rev Biophys.

[3] Plotkin SS, Onuchic JN (2002). Understanding protein folding with energy landscape theory – Part II: Quantitative aspects.. Q Rev Biophys.

[4] De Supinski BR, Schulz M, Bulatov VV, Cabot W, Chan B (2008). Bluegene/L applications: Parallelism on a massive scale.. Int J High Perform Comput Appl.

[5] Bowers KJ, Chow E, Xu H, Dror RO, Eastwood MP (2006). Algorithms for Molecular Dynamics Simulations on Commodity Clusters..

[6] Shirts M, Pande VS (2000). COMPUTING: Screen Savers of the World Unite!. Science.

[7] Hansmann UHE (1997). Parallel tempering algorithm for conformational studies of biological molecules.. Chem Phys Lett.

[8] Hukushima K, Nemoto K (1996). Exchange Monte Carlo method and application to spin glass simulations.. J Phys Soc Jpn.

[9] Sugita Y, Okamoto Y (1999). Replica-exchange molecular dynamics method for protein folding.. Chem Phys Lett.

[10] Oliveira CAFD, Hamelberg D, McCammon JA (2007). Estimating kinetic rates from accelerated molecular dynamics simulations: Alanine dipeptide in explicit solvent as a case study.. J Chem Phys.

[11] Dellago C, Bolhuis P, Csajka FS, Chandler D (1998). Transition path sampling and the calculation of rate constants.. J Chem Phys.

[12] Dellago C, Bolhuis P, Geissler P (2002). Transition path sampling.. Adv Chem Phys.

[13] van Erp T, Moroni D, Bolhuis PG (2003). A novel path sampling method for the calculation of rate constants.. J Chem Phys.

[14] Bolhuis PG (2003). Transition-path sampling of beta-hairpin folding.. Proc Natl Acad Sci U S A.

[15] Weinan E, Ren WQ, Vanden-Eijnden E (2005). Finite temperature string method for the study of rare events.. J Phys Chem B.

[16] Faradjian AK, Elber R (2004). Computing time scales from reaction coordinates by milestoning.. J Chem Phys.

[17] Neidigh JW, Fesinmeyer RM, Andersen NH (2002). Designing a 20-residue protein.. Nat Struct Biol.

[18] Qiu LL, Pabit SA, Roitberg AE, Hagen SJ (2002). Smaller and faster: The 20-residue Trp-cage protein folds in 4 µs.. J Am Chem Soc.

[19] Streicher WW, Makhatadze GI (2007). Unfolding thermodynamics of Trp-cage, a 20 residue miniprotein, studied by differential scanning calorimetry and circular dichroism spectroscopy.. Biochemistry.

[20] Ahmed Z, Beta IS, Mikhonin AV, Asher SA (2005). UV-resonance Raman thermal unfolding study of Trp-cage shows that it is not a simple two-state miniprotein.. J Am Chem Soc.

[21] Mok KH, Kuhn LT, Goez M, Day IJ, Lin JC (2007). A pre-existing hydrophobic collapse in the unfolded state of an ultrafast folding protein.. Nature.

[22] Neuweiler H, Doose S, Sauer M (2005). A microscopic view of miniprotein folding: Enhanced folding efficiency through formation of an intermediate.. Proc Natl Acad Sci U S A.

[23] Simmerling C, Strockbine B, Roitberg AE (2002). All-atom structure prediction and folding simulations of a stable protein.. J Am Chem Soc.

[24] Chowdhury S, Lee MC, Xiong GM, Duan Y (2003). Ab initio folding simulation of the Trp-cage mini-protein approaches NMR resolution.. J Mol Biol.

[25] Schug A, Herges T, Verma A, Lee KH, Wenzel W (2005). Comparison of Stochastic optimization methods for all-atom folding of the Trp-cage protein.. Chem Phys Chem.

[26] Schug A, Wenzel W, Hansmann U (2005). Energy landscape paving simulations of the trp-cage protein.. J Chem Phys.

[27] Schug A, Herges T, Wenzel W (2003). Reproducible protein folding with the stochastic tunneling method.. Phys Rev Lett.

[28] Ota M, Ikeguchi M, Kidera A (2004). Phylogeny of protein-folding trajectories reveals a unique pathway to native structure.. Proc Natl Acad Sci U S A.

[29] Pitera JW, Swope W (2003). Understanding folding and design: Replica-exchange simulations of “Trp-cage” fly miniproteins.. Proc Natl Acad Sci U S A.

[30] Zagrovic B, Pande V (2003). Solvent viscosity dependence of the folding rate of a small protein: Distributed computing study.. J Comput Chem.

[31] Zhou RH (2003). Trp-cage: Folding free energy landscape in explicit water.. Proc Natl Acad Sci U S A.

[32] Snow CD, Zagrovic B, Pande VS (2002). The Trp cage: Folding kinetics and unfolded state topology via molecular dynamics simulations.. J Am Chem Soc.

[33] Kentsis A, Gindin T, Mezei M, Osman R (2007). Calculation of the free energy and cooperativity of protein folding.. PLoS ONE.

[34] Paschek D, Nymeyer H, Garcia AE (2007). Replica exchange simulation of reversible folding/unfolding of the Trp-cage miniprotein in explicit solvent: On the structure and possible role of internal water.. J Struct Biol.

[35] Beck DAC, White GWN, Daggett V (2007). Exploring the energy landscape of protein folding using replica-exchange and conventional molecular dynamics simulations.. J Struct Biol.

[36] Juraszek J, Bolhuis PG (2006). Sampling the multiple folding mechanisms of Trp-cage in explicit solvent.. Proc Natl Acad Sci U S A.

[37] Juraszek J, Bolhuis PG (2008). Rate Constant and Reaction Coordinate of Trp-Cage Folding in Explicit Water.. Biophys J.

[38] Piana S, Laio A (2007). A bias-exchange approach to protein folding.. J Phys Chem B.

[39] Bussi G, Gervasio FL, Laio A, Parrinello M (2006). Free-energy landscape for beta hairpin folding from combined parallel tempering and metadynamics.. J Am Chem Soc.

[40] Piana S, Laio A, Marinelli F, Troys MV, Bourry D (2008). Predicting the effect of a point mutation on a protein fold: The villin and advillin headpieces and their Pro62Ala mutants.. J Mol Biol.

[41] Todorova N, Marinelli F, Piana S, Yarovsky I (2009). Exploring the Folding Free Energy Landscape of Insulin Using Bias Exchange Metadynamics.. J Phys Chem B.

[42] Leone V, Lattanzi G, Molteni C, Carloni P (2009). Mechanism of action of cyclophilin a explored by metadynamics simulations.. PLoS Comput Biol.

[43] Chodera JD, Singhal N, Pande VS, Dill KA, Swope WC (2007). Automatic discovery of metastable states for the construction of Markov models of macromolecular conformational dynamics.. J Chem Phys.

[44] Fischer A, Waldhausen S, Horenko I, Meerbach E, Schuette C (2007). Identification of Biomolecular conformations from incomplete torsion angle observations by hidden Markov models.. J Comput Chem.

[45] Jayachandran G, Vishal V, Pande VS (2006). Using massively parallel simulation and Markovian models to study protein folding: Examining the dynamics of the villin headpiece.. J Chem Phys.

[46] Horenko I, Dittmer E, Fischer A, Schuette C (2006). Automated model reduction for complex systems exhibiting metastability.. Multiscale Model Simul.

[47] Hummer G (2005). Position-dependent diffusion coefficients and free energies from Bayesian analysis of equilibrium and replica molecular dynamics simulations.. New J Phys.

[48] Buchete NV, Hummer G (2008). Coarse master equations for peptide folding dynamics.. J Phys Chem B.

[49] Kumar S, Rosenberg JM, Bouzida D, Swendsen RH, Kollman PA (1995). Multidimensional freeenergy calculations using the weighted histogram analysis method.. J Comput Chem.

[50] Bicout DJ, Szabo A (1998). Electron transfer reaction dynamics in non-Debye solvents.. J Chem Phys.

[51] Bortz AB, Kalos MH, Lebowitz JL (1975). New algorithm for monte-carlo simulation of ising spin systems.. J Comput Phys.

[52] Voter AF, Sickafus KE, Kotomin EA (2005). Introduction to the Kinetic Monte Carlo Method.. Radiation Effects in Solids..

[53] Enright AJ, Dongen SV, Ouzounis CA (2002). An efficient algorithm for large-scale detection of protein families.. Nucleic Acids Res.

[54] Gfeller D, Rios PDL, Caflisch A, Rao F (2007). Complex network analysis of free-energy landscapes.. Proc Natl Acad Sci U S A.

[55] Laio A, Gervasio FL (2008). Metadynamics: a method to simulate rare events and reconstruct the free energy in biophysics, chemistry and material science.. Rep Prog Phys.

[56] Bussi G, Laio A, Parrinello M (2006). Equilibrium free energies from nonequilibrium metadynamics.. Phys Rev Lett.

[57] Daura X, Gademann K, Jaun B, Seebach D, van Gunsteren WF (1999). Peptide folding: When simulation meets experiment.. Angew Chem-Int Edit.

[58] Micheletti C, Laio A, Parrinello M (2004). Reconstructing the density of states by history-dependent metadynamics.. Phys Rev Lett.

[59] Lindahl E, Hess B, van der Spoel D (2001). GROMACS 3.0: a package for molecular simulation and trajectory analysis.. J Mol Model.

[60] Berendsen HJC, der Spoel DV, Vandrunen R (1995). GROMACS - a message-passing parallel molecular-dynamics implementation.. Comput Phys Commun.

[61] Duan Y, Wu C, Chowdhury S, Lee MC, Xiong GM (2003). A point-charge force field for molecular mechanics simulations of proteins based on condensed-phase quantum mechanical calculations.. J Comput Chem.

[62] Jorgensen WL, Chandrasekhar J, Madura JD, Impey RW, Klein ML (1983). Comparison of simple potential functions for simulating liquid water.. J Chem Phys.

[63] Hess B, Bekker H, Berendsen HJC, Fraaije GEMJ (1997). Lincs: A linear constraint solver for molecular simulations.. J Comput Chem.

[64] Miyamoto S, Kollman PA (1992). An analytical version of the SHAKE and RATTLE algorithms for rigid water models.. J Comput Chem.

[65] Darden TA, York D (1993). Particle mesh ewald - an n.log(n) method for ewald sums in large systems.. J Chem Phys.

[66] Essman U, Perera L, Berkowitz ML, Darden TA, Lee H (1995). A smooth particle mesh ewald method.. J Chem Phys.

[67] Berendsen HJC, Postma JPM, Gusteren WFV, Nola AD, Haak JR (1984). Molecular dynamics with coupling to an external bath.. J Chem Phys.

[68] Hornak V, Abel R, Okur A, Strockbine B, Roitberg A (2006). Comparison of multiple amber force fields and development of improved protein backbone parameters.. Proteins.

[69] Graf J, Nguyen PH, Stock G, Schwalbe H (2007). Structure and dynamics of the homologous series of alanine peptides: A joint molecular dynamics/NMR study.. J Am Chem Soc.

[70] Mu YG, Kosov DS, Stock G (2003). Conformational dynamics of trialanine in water. 2. Comparison of AMBER, CHARMM, GROMOS, and OPLS force fields to NMR and infrared experiments.. J Phys Chem B.

[71] Xu X, Moon S, Case D (2005). SHIFTS Program.. Department Molecular Biology, The Scripps Research Institute.

[72] Eisenberg D, Mclachlan A (1986). Solvation energy in protein folding and binding.. Nature.

[73] Woutersen S, Hamm P (2000). Structure determination of trialanine in water using polarization sensitive two-dimensional vibrational spectroscopy.. J Phys Chem B.

[74] Schweitzer-Stenner R, Eker F, Huang Q, Griebenow K (2001). Dihedral angles of trialanine in D2O determined by combining FTIR and polarized visible Raman spectroscopy.. J Am Chem Soc.

[75] Schweitzer-Stenner R (2002). Dihedral angles of tripeptides in solution directly determined by polarized Raman and FTIR spectroscopy.. Biophys J.

[76] Roder H, Maki K, Cheng H (2006). Early events in protein folding explored by rapid mixing methods.. Chem Rev.

[77] Rhee Y, Sorin E, Jayachandran G, Lindahl E, Pande V (2004). Simulations of the role of water in the protein-folding mechanism.. Proc Natl Acad Sci U S A.

[78] Shen M, Freed K (2002). Long time dynamics of met-enkephalin: Comparison of explicit and implicit solvent models.. Biophys J.

[79] Bunagan M, Yang X, Saven J, Gai F (2006). Ultrafast folding of a computationally designed Trpcage mutant: Trp(2)-cage.. J Phys Chem B.

[80] Barua B, Lin JC, Williams VD, Kummler P, Neidigh JW (2008). The Trp-cage: optimizing the stability of a globular miniprotein.. Protein Eng Des Sel.

[81] Wallace A, Laskowski R, Thornton J (1995). LIGPLOT - A Program to generate schematic diagrams of protein ligand interactions.. Protein Eng.

[82] Hirst JD, B CL (1994). Helicity, circular dichroism and molecular dynamics of proteins.. J Mol Biol.

