# Supplementary figures and images for: A Kinetic Model of Trp-Cage Folding from Multiple Biased Molecular Dynamics Simulations
Source: PLoS Comput Biol. 2009 Aug 7;5(8):e1000452. doi: 10.1371/journal.pcbi.1000452 (PMC2711228; doi:10.1371/journal.pcbi.1000452)

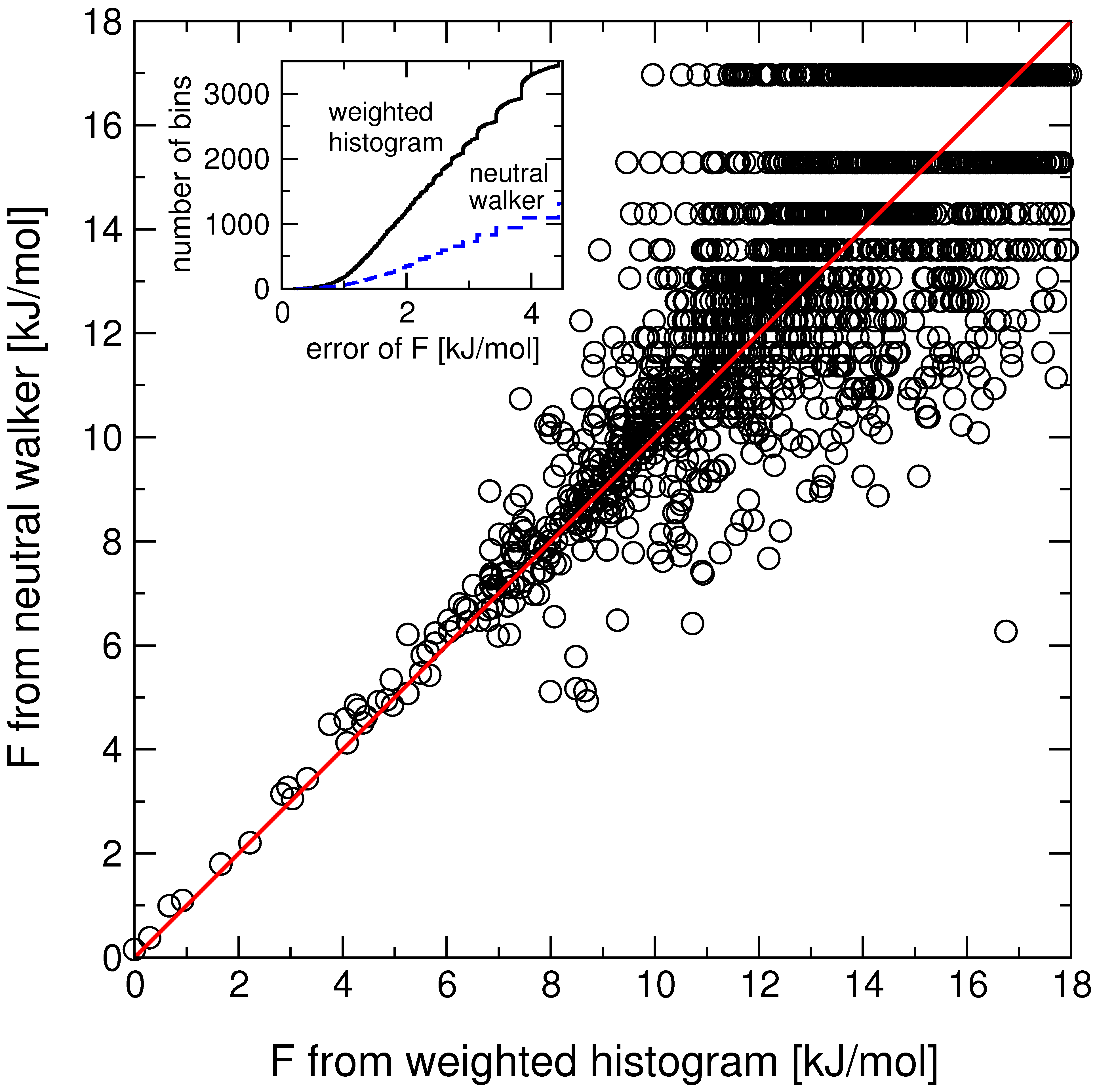

Supplement: Figure S3 — Correlation between free energies of neutral walker and WHAM for Trp-cage. Correlation between the bins free energy evaluated using the approach described in the Methods section and using the neutral walker ensemble at T = 298 K. Inset: cumulative number of bins with an error smaller than the value reported in abscissas. The error is estimated using Eq. 8. The value of g entering this equation is estimated from the correlation time of the bin occupancies and is equal to 10 ps. (0.39 MB TIF) [file pcbi.1000452.s008.tif]

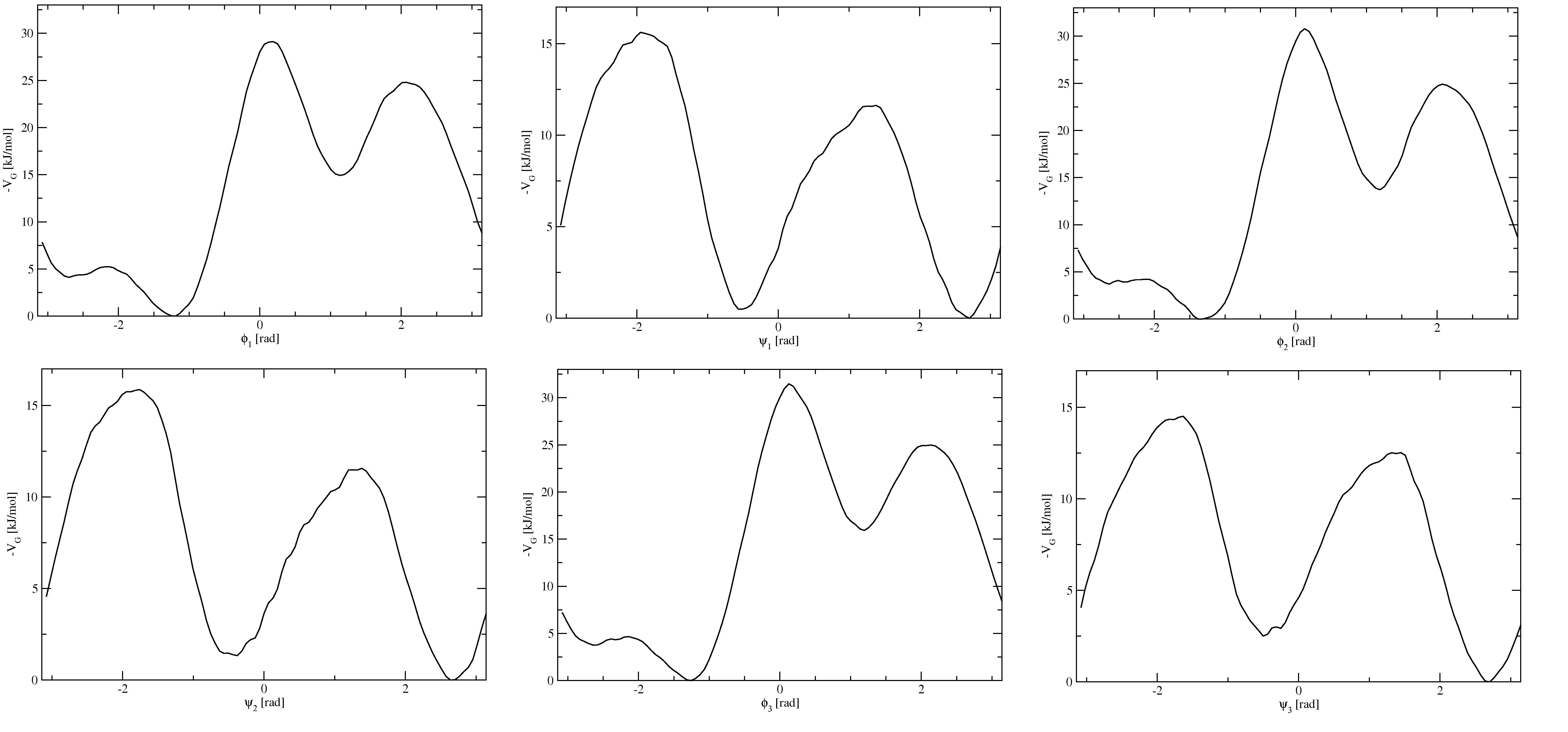

Supplement: Figure S5 — Free energy profiles of Ala3 along the six backbone dihedral angles. The profiles are calculated using eq. 2 on the last 10 ns of a 30 ns BE simulation. (0.15 MB TIF) [file pcbi.1000452.s010.tif]

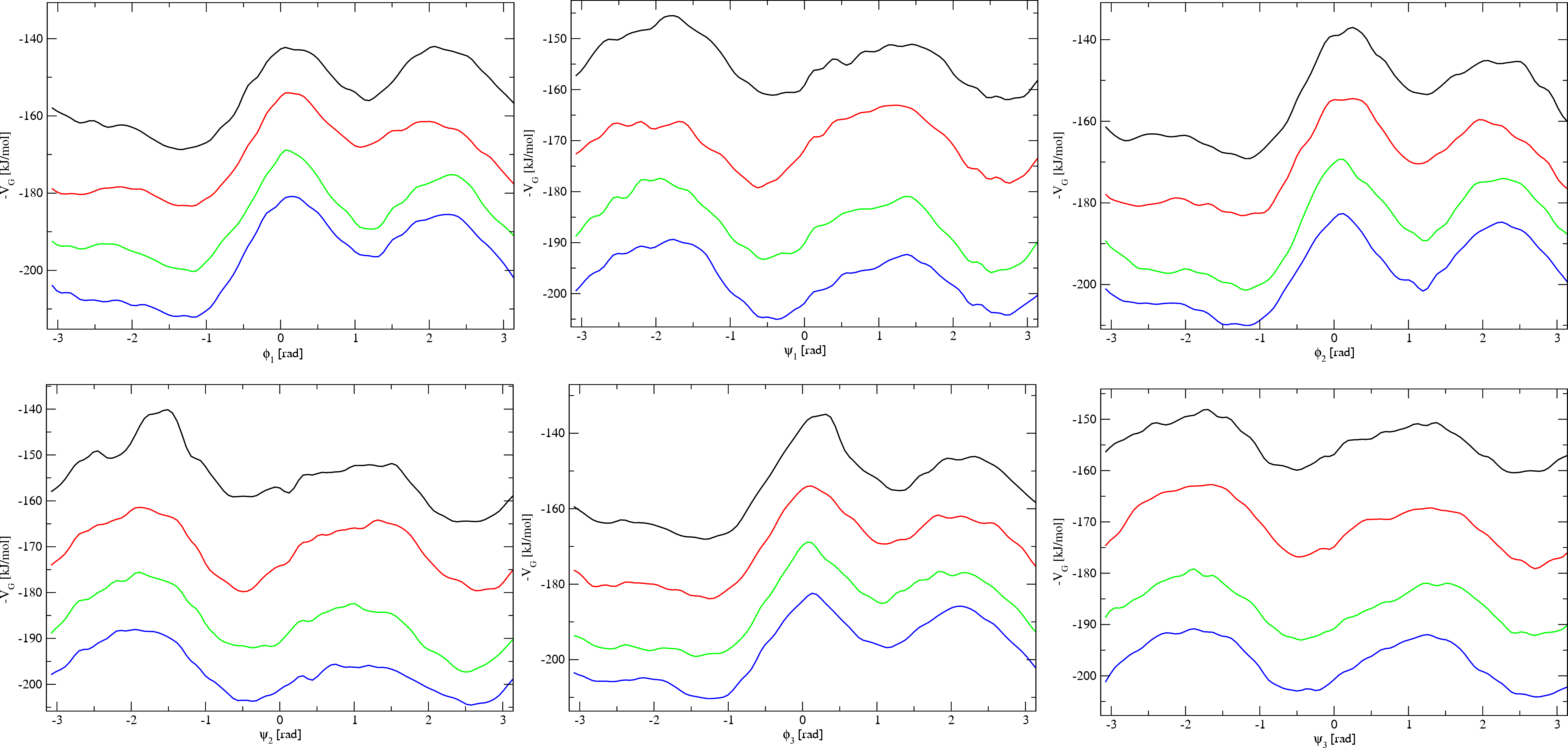

Supplement: Figure S6 — Free energy profiles as a function of time for Ala3 obtained with a 30 ns BE simulation. −VG is reported for each backbone dihedral angle at several times after the filling time. Each time is represented with a different color: black (10 ns), red (11 ns), green (12 ns) and blue (13 ns). The parallel growth in time of the metadynamics bias potential is evident from the picture. (0.36 MB TIF) [file pcbi.1000452.s011.tif]

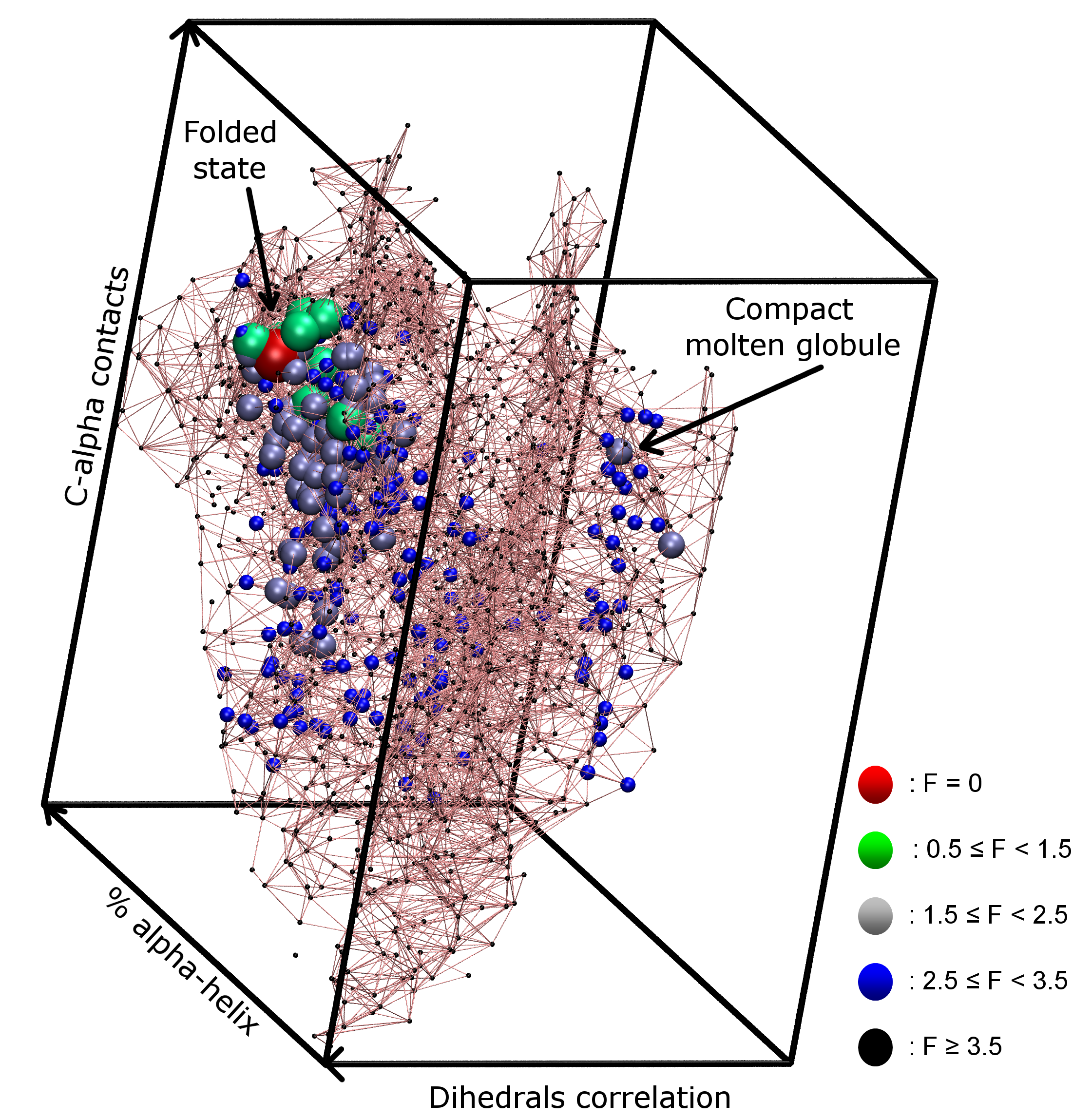

Supplement: Figure S8 — Bins network topology at T = 298 K projected on three dimensions: Cα contacts, dihedral correlations and α-helix fraction. Each bin is represented as a sphere whose dimension and color is associated with the free energy (kcal/mol). The location of the folded state and the molten globule (cluster 5) lowest free energy bins are indicated in the figure. (3.07 MB TIF) [file pcbi.1000452.s013.tif]
