# Supplementary material for: A Kinetic Model of Trp-Cage Folding from Multiple Biased Molecular Dynamics Simulations
Source: PLoS Comput Biol. 2009 Aug 7;5(8):e1000452. doi: 10.1371/journal.pcbi.1000452 (PMC2711228; doi:10.1371/journal.pcbi.1000452)
Supplement: Text S1 — Diffusion matrix tables and correspoding rates. (0.07 MB PDF) [file pcbi.1000452.s014.pdf]

A kinetic model of Trp-cage folding from multiple biased molecular dynamics simulations: Text S1

Table 1: **Diffusion matrix of Ala$_3$**. An MD trajectory of 60 ns is employed, using a time lag of 14 ps. The elements of the diffusion matrix are expressed in rad^2/ps. The position dependence of the matrix and the statistical uncertainty give a total estimated error of 20%.

	ϕ_1	ψ_1	ϕ_2	ψ_2	ϕ_3	ψ_3
ϕ_1	0.040	0.000	0.000	0.000	0.000	0.000
ψ_1	0.000	0.037	-0.018	0.000	0.000	0.000
ϕ_2	0.000	-0.018	-0.034	0.000	0.000	0.000
ψ_2	0.000	0.000	0.000	0.034	-0.014	0.000
ϕ_3	0.000	0.000	0.000	-0.014	0.040	0.000
ψ_3	0.000	0.000	0.000	0.000	0.000	0.038

Table 2: **Diffusion matrix of Trp-cage**. five MD trajectories for a cumulative time of 500 ns are employed, using a time lag of 12 ns. Units are 1/ns.

	$CV1$	$CV2$	$CV3$	$CV4$	$CV5$
$CV1$	0.445	0.263	0.010	0.012	-0.010
$CV2$	0.263	2.725	0.530	0.034	-0.025
$CV3$	0.010	0.530	0.300	-0.005	-0.015
$CV4$	0.012	0.034	-0.005	0.037	-0.003
$CV5$	-0.010	-0.025	-0.015	-0.003	0.040

Table 3: **Diffusion matrix of Trp-cage close to the folded state (cluster 1)**. A MD trajectory of 80 ns starting from the folded state and remaining close to it is employed, using a time lag of 12 ns. Units are 1/ns. The statistical uncertainty on each element has been calculated by dividing the overall trajectory in 3 pieces and evaluating the diffusion matrix independently in each part. The simulated T-jump experiment (see manuscript) performed using this diffusion matrix gives a relaxation time of 2322 ns

	$CV1$	$CV2$	$CV3$	$CV4$	$CV5$
$CV1$	0.867±0.180	-0.060±0.001	-0.026±0.002	0.0009±0.0001	-0.009±0.0003
$CV2$	-0.060±0.001	3.027±0.474	0.660±0.155	0.010±0.002	-0.020±0.004
$CV3$	-0.026±0.002	0.660±0.155	0.343±0.045	0.00040±0.00003	0.002±0.004
$CV4$	0.0009±0.0001	0.010±0.002	0.00040±0.00003	0.073±0.003	-0.005±0.002
$CV5$	-0.0090±0.0003	-0.020±0.004	0.002±0.004	-0.005±0.002	0.028±0.003

Table 4: **Diffusion matrix of Trp-cage in cluster 2**. A MD trajectory of 200 ns exploring cluster 2 is employed, using a time lag of 12 ns. Units are 1/ns. The statistical uncertainty on each element has been calculated by dividing the overall trajectory in 3 pieces and evaluating the diffusion matrix independently in each part. The simulated T-jump experiment (see manuscript) performed using this diffusion matrix gives a relaxation time of 2194 ns

	$CV1$	$CV2$	$CV3$	$CV4$	$CV5$
$CV1$	0.400±0.066	0.324±0.097	0.014±0.007	0.013±0.007	-0.010±0.003
$CV2$	0.324±0.097	2.745±0.473	0.526±0.027	0.035±0.008	-0.032±0.005
$CV3$	0.014±0.007	0.526±0.027	0.310±0.004	-0.006±0.004	-0.017±0.004
$CV4$	0.013±0.00	0.035±0.008	-0.006±0.004	0.032±0.008	-0.003±0.003
$CV5$	-0.010±0.003	-0.032±0.005	-0.017±0.004	-0.003±0.003	0.046±0.003

Table 5: **Diffusion matrix of Trp-cage in cluster 3**. A MD trajectory of 90 ns exploring cluster 3 is employed, using a time lag of 12 ns. Units are 1/ns. The statistical uncertainty on each element has been calculated by dividing the overall trajectory in 3 pieces and evaluating the diffusion matrix independently in each part. The simulated T-jump experiment (see manuscript) performed using this diffusion matrix gives a relaxation time of 3052 ns

	$CV1$	$CV2$	$CV3$	$CV4$	$CV5$
$CV1$	0.410±0.075	0.320±0.068	0.017±0.005	0.023±0.001	-0.008±0.001
$CV2$	0.320±0.068	1.630±0.055	0.500±0.080	0.0470±0.002	-0.030±0.007
$CV3$	0.017±0.005	0.500±0.080	0.286±0.037	-0.008±0.002	-0.0222±0.0008
$CV4$	0.023±0.001	0.0470±0.002	-0.008±0.002	0.033±0.004	-0.0060±0.0005
$CV5$	-0.008±0.001	-0.030±0.007	-0.0222±0.0008	-0.0060±0.0005	0.036±0.002

Table 6: **Diffusion matrix of Trp-cage in extended states and cluster 4**. A MD trajectory of 70 ns exploring cluster 4 is employed, using a time lag of 12 ns. Units are 1/ns. The statistical uncertainty on each element has been calculated by dividing the overall trajectory in 3 pieces and evaluating the diffusion matrix independently in each part. The simulated T-jump experiment (see manuscript) performed using this diffusion matrix gives a relaxation time of 3340 ns

	$CV1$	$CV2$	$CV3$	$CV4$	$CV5$
$CV1$	0.307±0.001	0.510±0.003	0.030±0.002	0.018±0.001	-0.00610±0.00001
$CV2$	0.510±0.003	2.887±0.270	0.533±0.016	0.050±0.005	-0.030±0.012
$CV3$	0.030±0.002	0.533±0.016	0.482±0.077	-0.0114±0.0002	-0.0120±0.0001
$CV4$	0.018±0.001	0.050±0.005	-0.0114±0.0002	0.050±0.003	-0.0064±0.0005
$CV5$	-0.00610±0.00001	-0.030±0.012	-0.0120±0.0001	-0.0064±0.0005	0.010±0.002

Table 7: **Diffusion matrix of Trp-cage in cluster 5 (molten globule)**. A MD trajectory of 65 ns exploring cluster 5 is employed, using a time lag of 12 ns. Units are 1/ns. The statistical uncertainty on each element has been calculated by dividing the overall trajectory in 3 pieces and evaluating the diffusion matrix independently in each part. The simulated T-jump experiment (see manuscript) performed using this diffusion matrix gives a relaxation time of 2148 ns

	$CV1$	$CV2$	$CV3$	$CV4$	$CV5$
$CV1$	0.362±0.007	0.156±0.002	0.004±0.00006	0.004±0.00005	-0.011±0.0001
$CV2$	0.156±0.002	2.875±0.040	0.541±0.010	0.0180±0.0002	-0.023±0.0003
$CV3$	0.004±0.00006	0.541±0.010	0.203±0.004	-0.000140±0.000002	-0.0130±0.0005
$CV4$	0.004±0.00005	0.0180±0.0002	-0.000140±0.000002	0.0270±0.0003	0.00151±0.00002
$CV5$	-0.011±0.0001	-0.023±0.0003	-0.0130±0.0005	0.00151±0.00002	0.040±0.002
